# Economic Evaluation of a General Hospital Unit for Older People with Delirium and Dementia (TEAM Randomised Controlled Trial)

**DOI:** 10.1371/journal.pone.0140662

**Published:** 2015-12-18

**Authors:** Lukasz Tanajewski, Matthew Franklin, Georgios Gkountouras, Vladislav Berdunov, Rowan H. Harwood, Sarah E. Goldberg, Lucy E. Bradshaw, John R. F. Gladman, Rachel A. Elliott

**Affiliations:** 1 School of Pharmacy, University of Nottingham, Nottingham, United Kingdom; 2 Health Care of Older People, Nottingham University Hospitals NHS Trust, Queens Medical Centre, Nottingham, United Kingdom; 3 School of Health Sciences, University of Nottingham, Nottingham, United Kingdom; 4 Division of Rehabilitation and Ageing, University of Nottingham, Nottingham, United Kingdom; University of Glasgow, UNITED KINGDOM

## Abstract

**Background:**

One in three hospital acute medical admissions is of an older person with cognitive impairment. Their outcomes are poor and the quality of their care in hospital has been criticised. A specialist unit to care for older people with delirium and dementia (the Medical and Mental Health Unit, MMHU) was developed and then tested in a randomised controlled trial where it delivered significantly higher quality of, and satisfaction with, care, but no significant benefits in terms of health status outcomes at three months.

**Objective:**

To examine the cost-effectiveness of the MMHU for older people with delirium and dementia in general hospitals, compared with standard care.

**Methods:**

Six hundred participants aged over 65 admitted for acute medical care, identified on admission as cognitively impaired, were randomised to the MMHU or to standard care on acute geriatric or general medical wards. Cost per quality adjusted life year (QALY) gained, at 3-month follow-up, was assessed in trial-based economic evaluation (599/600 participants, intervention: 309). Multiple imputation and complete-case sample analyses were employed to deal with missing QALY data (55%).

**Results:**

The total adjusted health and social care costs, including direct costs of the intervention, at 3 months was £7714 and £7862 for MMHU and standard care groups, respectively (difference -£149 (95% confidence interval [CI]: -298, 4)). The difference in QALYs gained was 0.001 (95% CI: -0.006, 0.008). The probability that the intervention was dominant was 58%, and the probability that it was cost-saving with QALY loss was 39%. At £20,000/QALY threshold, the probability of cost-effectiveness was 94%, falling to 59% when cost-saving QALY loss cases were excluded.

**Conclusions:**

The MMHU was strongly cost-effective using usual criteria, although considerably less so when the less acceptable situation with QALY loss and cost savings were excluded. Nevertheless, this model of care is worthy of further evaluation.

**Trial Registration:**

ClinicalTrials.gov NCT01136148

## Introduction

### Background

About 50% of people over the age of 65 in general hospitals have delirium, dementia or both, representing one in three hospital acute medical admissions. [1–3] Various models have been proposed to provide for their particular needs. [3–5] The National Dementia Strategy for England promotes old age liaison psychiatry services, [4] although it is unclear of what such services should comprise, how they facilitate high quality care, and there is no firm evidence of their cost-effectiveness. [5] We developed an alternative model—a specialist unit in a general hospital to care for people with delirium and dementia (the Medical and Mental Health Unit (MMHU)). [6] Its development aimed to reflect best practice in dementia and delirium care taking into account the published literature, [6,7] [8] and expert opinion from clinicians working in the field. It was tested in a randomised controlled trial (Trial of an Elderly Acute care Medical and mental health unit (TEAM)), [7,8] which showed that the quality of care was higher, as judged by direct observation and carer satisfaction, but benefits in health status outcomes at three months were small and not statistically significant [8]. There are no other robust studies of these types of specialist units and the cost and economic implications of this model of care are not yet known.

This analysis compared the costs and cost-effectiveness of the MMHU with those of standard care, from the perspective of the National Health Service and publically funded personal social care. The trial-based economic evaluation is reported in accordance with the CHEERS Statement (S1 Appendix).

### Medical and Mental Health Unit and standard care wards

An existing 28-bed acute geriatric medical ward, including its ward-based staff, was converted to a specialist unit, MMHU, where only older patients with cognitive impairment were admitted. Five main areas of enhancement (described in depth elsewhere [6]) were: 1) Additional specialist mental health staff were employed (mental health nurses and occupational therapist along with additional support from physiotherapy, speech and language therapy, psychiatry and geriatric medicine), including three healthcare assistants working as activity coordinators; 2) Staff training in recognition and management of delirium and dementia and the delivery of person-centred dementia care; 3) A programme of organised therapeutic and diversionary activities; 4) The environment was made more appropriate for people with cognitive impairment; 5) A proactive and inclusive approach to family carers was promoted.

Standard care wards included five acute geriatric medical wards, and six general (internal) medical wards. Practice on geriatric medical wards was based on the principles of comprehensive geriatric assessment, [9] and staff had general experience in the management of delirium and dementia. Mental health support was provided, on request, from visiting psychiatrists. There was no dedicated old age liaison psychiatry service at that time. None of the MMHU enhancements listed and described above was routine on standard care wards.

### TEAM trial

A randomised controlled trial, Trial of an Elderly Acute care Medical and mental health unit (TEAM), was conducted. [8] The trial protocol (S1 Protocol) was published, [7] and the full report on the trial, including recruitment flow chart, is available elsewhere as an open-access article.[8]

The protocol for the TEAM study was given a favourable opinion by the Nottingham 1 Research Ethics Committee (reference 10/H0403/1). Recruitment of patient participants followed the requirements of the English Mental Capacity Act (2005) and was approved by the research ethics committee. After allocation to a ward, research staff identified patients who had been randomised, discussed the study with them and assessed them for capacity to give consent to take part in the study. Those with capacity who were willing to participate were asked to give written consent. Most potential participants lacked capacity, in which case an informal carer was asked to give written agreement to participation. If there was no available carer, the nurse in charge of the ward was asked to act as a "professional consultee".

Patients were recruited who had been admitted for acute medical care to a single large teaching hospital. Participants were aged over 65, and identified by admissions unit physicians as being ‘confused’. We used the term ‘confused’ as there is considerable overlap between delirium and dementia in this population, [3] and dementia is often undiagnosed in the community and hospital. [3,10] Suitable patients identified on the hospital’s medical admissions unit were entered onto a computerised screening log and, if a bed was available on the MMHU, randomised 1:1 between the MMHU and standard care in a permuted block design, stratified for previous care home residence. Randomised patients who were readmitted were assigned their original allocation. Regardless of allocation, patients had access to standard medical and mental health services, rehabilitation, intermediate and social care. Baseline clinical data was collected from the patient, family members, or other informal or professional carers by interview with a researcher. Outcomes were ascertained at interviews at home 90 days (±7 days) after randomisation by researchers not involved in recruitment or baseline data collection, and blind to allocation.

Between July 2010 and December 2011, 310 patients were recruited from the MMHU and 290 from standard care. One patient in the MMHU arm was lost to follow-up (moved away from the geographical area). A professional consultee was involved in the recruitment of 30 MMHU and 31 standard care participants, as allowed by English mental capacity law when a patient lacking mental capacity has no family or friends to advocate for them. Baseline characteristics of the population and clinical effectiveness outcomes have been reported previously. [8] In short, there was no statistically significant difference between settings in the trial primary outcome, days spent at home (median 51 vs 45 days; 95% confidence interval [CI] for difference -12 to 24; *p* = 0.3); median index hospital stay was 11 vs 11 days, mortality 22% vs 25% (95% CI for difference: -9%, 4%), readmission 32% vs 35% (95% CI for difference: -10%, 5%), and new care home admission 20% vs 28% (95% CI for difference: -16%, 0), for the MMHU and standard care, respectively. Participants on the MMHU spent significantly more time with positive mood or engagement (79% vs 68%; 95% CI for difference: 2%, 20%; *p* = 0.03), and experienced more staff interactions that addressed emotional and psychological needs (median 4 vs 1 per observation; p<0.001). More family carers were satisfied with care (overall 91% vs 83%; 95% CI for difference: 2%, 15%; *p* = 0.004), and severe dissatisfaction was reduced (5% vs 10%; 95% CI for difference: -10%, 0; *p* = 0.05). [8]

## Methods

### Health effects

The health outcome for the cost-effectiveness analysis was quality-adjusted life year (QALY) gained, constructing utility values from the 3-level EuroQol-5D (EQ-5D-3L) [11] with societal weights. [12] We used EQ-5D utility measure in this economic evaluation because of its relevance for UK policy makers, particularly the National Institute for Health and Care Excellence (NICE). [13] Patient-reported EQ-5D valuations at baseline and 90-day follow up (measuring health state on a scale in which 0 and 1 represent death and full health, respectively) were applied to estimate QALYs gained, assuming baseline utility until date of death for a patient dead at follow up. Hence, a patient’s QALYs gained were calculated as the area under curve using linear interpolation between EQ-5D measurement points, and health outcome was summarised into a single index. Due to the nature of the population studied, 55% of participants had missing data for self-reported EQ-5D. Other health status variables [8] were used to impute values in these cases, including proxy completed EQ-5D, dementia-related quality-of-life at follow up (DEMQOL [14]), behavioural and psychological symptoms (Neuro-Psychiatric Inventory (NPI) [15]), and dependency in personal activities of daily living (Barthel ADL [16]).

### Costs

#### Costs of delivering the MMHU intervention

The MMHU intervention cost was calculated as the additional MMHU staffing cost compared with standard care on a general or geriatric ward–additional staff employed on MMHU and associated costs are presented in Table 1. Staff salary levels were based on salary levels from NHS pay scales 2011/12. [17] In order to estimate the cost of staff involved in direct patient care, as opposed to other activities such as general management and training, salary costs were adjusted by the proportion of time spent on patient care on the ward. For instance, the occupational therapist, mental health nurse and consultant spent two-thirds of their time on direct patient care so their total annual cost was multiplied by 0.67. The total additional staffing cost was allocated on an individual patient basis (for patients recruited to the MMHU arm of the trial), assuming 100% bed occupancy on MMHU (28 beds), by multiplying the per-bed-day additional MMHU cost by the individual patient’s length of stay on MMHU–calculations are presented in Table 1.

**Table 1:** Derivation of MMHU intervention cost.

Category (NHS salary band)	N	Annual salary (£)^a^	On-costs (£)^b^	Total annual cost (£)	Ward time adjustment^c^	Adjusted total annual cost (£)^c^
Occupational therapist (band 7)	1	35184	8268	43452	0.67 spent on ward	29113
Healthcare assistant (band 2)	2	30473	6625	37098	100% on ward	37098
Mental health nurse (band 7)	1	35184	8898	44082	0.67 spent on ward	29535
Mental health nurse (band 5)	2	48143	11618	59761	100% on ward	59761
Speech and language therapist (band 6)	0.1	2946	692	3639	100% on ward	3639
Activity coordinator (band 2)	3	45710	9937	55646	100% on ward	55646
Consultant (MC58)	0.3	26811	7226	34037	0.67 spent on ward	22805
Physiotherapy (band 6)	0.5	14732	3462	18194	100% on ward	18194
**Total annual MMHU additional cost**				**295909**		**255791**
Additional cost per-day^d^						700
Additional cost per-bed-day^e^						25
***Mean per-patient intervention cost (full-sample)*** ^f^						***368 (95% CI*: *334*, *410)***

NB: all figures presented are rounded to 0 decimal places.

^a^Annual salary based on proportion of time employed for working on MMHU; annual staffing and salary information from ward proposal, based on 2011/12 FY NHS pay scales mid-point salary levels; consultant salary was based on threshold 6 of pay scale MC58 for 2011/12 FY.

^b^Salary on-costs taken from PSSRU 2011/12.

^c^Total cost adjusted based on time spent on the MMHU during the trial period–time spent by professional on training staff and management not included in ward time adjustment.

^d^Calculated as: £255790.55/365.25 = £700.32.

^e^Calculated, assuming 100% occupancy (28 beds), as: £700.32/28 = £25.01.

^f^Calculated as mean per-patient MMHU additional cost for participants recruited to the MMHU arm of the trial (309 patients in the full sample CEA), for whom mean length of stay on MMHU was 14.73 days (95% confidence interval [CI]: 13.35, 16.37): £25.01∙14.73 = £368.45. MMHU intervention cost is calculated on an individual patient basis, by multiplying per-bed-day MMHU additional cost (£25.01) by the patient’s length of stay on MMHU. Mean per-patient intervention costs for the complete-case CEA dataset is presented in Table 4.

#### Health and social care resource-use data

Most health and social care services now use electronic administrative record systems to record patient care. [18] Approvals were gained to obtain electronic administrative record systems data from hospitals, social care, general practices (GP), ambulance services, and mental healthcare. Data were collected for three months post-hospital admission and one year pre-admission (July 2009 –March 2012). Based on our previous research, [18] extensive fieldwork was completed with the included agencies to derive parameters covering resource-use (details in S2 Appendix).

Hospital care data (day-case, inpatient, outpatient and intensive care) were obtained from two patient administration systems for patients that attended five hospitals in the Nottingham area. Primary care resource-use data were obtained from Nottingham City and Nottinghamshire County GP practices. Of 107 GP practices serving our cohort, data were obtained from 72 practices (468/599 participants), coming from five electronic administrative record systems: SystmOne, 220 (47%); EMIS LV, 196 (42%); EMIS Web, 34 (7%); Synergy, 13 (3%); and EMIS PCS, 5 (1%), and were anonymised at the GP practice. Ambulance service resource-use was extracted from the Caller Aided Despatch (CAD) IT service. The CAD system was cross-referenced with paper-based Patient Record Forms to identify participants (using participant name and place of pick-up). Data from mental healthcare services for older people were provided by the Nottinghamshire Healthcare Trust data via the RiO system. [19] Social care services within two different catchment areas (City and County), operating two different electronic systems, were identified. Services consisted of contacts and assessments, and care plans. Care plans included home, day, residential and telecare, housing and meals at home services.

#### Patient-level cost

Unit costs for primary care services were applied based on time taken to perform each task using time assumptions obtained from PSSRU 2011/12, [17] empirical literature, or expert opinion, and mid-point yearly salary estimations taken from the NHS “Agenda for Change” pay rates. [20] Unit costs of hospital care were applied using national reference costs according to Healthcare Resource Group (HRG) case-mix. Inpatient spell costs were adjusted for length-of-stay using standard excess bed day costs. Unit costs for other services were obtained from PSSRU, standard Department of Health costs and other reference costs for the 2011/12 financial year. The detailed costing methods are described elsewhere, [18] the sources of unit costs are presented in S2 Appendix and the HRG codes used to derive costs are presented in S3 Appendix. Due to the high number of different parameters and unit costs used to calculate patient-level cost (an example of which is provided in S3 Appendix for the codes used to assign unit costs to hospital resource-use), only a brief overview of the other costs are described below.

Unit costs were combined with resource-use to generate patient-level costs. Patient-level costs from all health and social care services incurred during the trial were calculated for all trial participants who remained in the study at 90-day follow-up (patients who died during the study were not classed as ‘withdrawn’).

### Cost-effectiveness analysis

The economic evaluation adopted a health and social services perspective. The incremental cost-effectiveness ratio (ICER) generated by the MMHU over standard care was calculated using the following equation:

$$ICER=\frac{Cost_{MMHU}-Cost_{SC}}{QALY_{MMHU}-QALY_{SC}},$$

where *Cost*_*MMHU*_ (*Cost*_*SC*_) and *QALY*_*MMHU*_ (*QALY*_*SC*_) are mean cost and QALYs gained in the MMHU (standard care) group, respectively. Patient cost and QALYs were adjusted by baseline characteristics using regression methods, including one year pre-admission healthcare costs as a covariate when modelling costs. Pairwise bootstrapping with replacement was employed for adjusted patient costs and QALYs, using 5000 replications. The resultant incremental costs and outcomes were plotted on a cost-effectiveness plane. [21] To investigate uncertainty around the ICER, cost-effectiveness acceptability curves (CEACs) [22,23] based on ceiling ratios were constructed. These (standard) CEACs represent probability of cost-effectiveness for a given willingness to pay (WTP) for QALY gain, equal to willingness to accept (WTA) QALY loss, that is, WTA/WTP ratio equal to 1. [24]

Sensitivity analysis to capture WTA/WTP disparity was conducted. Probability of cost-effectiveness for a £20,000 WTP threshold in relation to WTA/WTP ratio was investigated, [24] to account for the notion that QALY gains at additional cost (WTP) may be more acceptable for decision makers, when compared to cost savings and QALY losses (WTA), as suggested in the health and behavioral economics literature. [24–26] Conservatively, WTA/WTP ratio between 1 and infinity [24], corresponding to WTA threshold between £20,000 and infinity for accepting QALY loss, respectively (SW quadrant of cost-effectiveness plane), was assumed in the sensitivity analysis. Namely, the WTA/WTP ratio, *r*, *r* ≥ 1, reflected the proportion that, paying £20,000 *maximum* for QALY gain (NE quadrant of cost-effectiveness plane), QALY loss was accepted for *minimum r* ∙ £20,000 (SW quadrant of cost-effectiveness plane).

The analyses were performed using STATA version 12 [27] and Microsoft Excel 2010.

#### Missing data

In the case of 90-day (trial) cost data, only primary care data were missing (131/599 (21.9%) patients). One year pre-admission healthcare cost was missing for inpatient care (2/599 (0.3%)) and for primary care (155/599 (25.9%)).

Missing data for patient-reported EQ-5D are: 195/599 (32.6%) baseline EQ-5D, 209/599 (34.9%) follow-up EQ-5D, resulting in QALYs obtained for 272/599 (45.4%) patients, including 62 (MMHU: 30) dead at follow up.

No statistically significant differences in the proportions of missing EQ-5D and QALYs values between MMHU and standard care groups were observed: 92/309 (29.8%) vs. 103/290 (35.5%), *p* = 0.13, for baseline EQ-5D; 113/309 (36.6%) vs 96/290 (33.1%), *p* = 0.37, for follow-up EQ-5D; and 170/309 (55.0%) vs 157/290 (54.1%), *p* = 0.83, for QALYs. Furthermore, for primary care cost and for other health measurement variables of interest, the differences in the proportions of missing values between arms were non-significant; the percentage of missing values in the two arms was similar apart from proxy completed EQ-5D and follow-up Barthel ADL index (see Table A in S4 Appendix).

Missing values for cost, EQ-5D, and for other variables, were assumed to be missing at random (MAR). Given no imbalance in proportions of missing values between randomised groups (as shown above and in Table A in S4 Appendix) and predictors of missing values for EQ-5D (and for other health status variables) identified among variables with complete data (age, number of medical conditions, and permanent care home residence at baseline—see Tables B-F in S4 Appendix for details) the MAR assumption seemed to be plausible. Hence, the multiple imputation approach was applied to deal with missing data in cost-effectiveness analysis.

Missing values for cost, EQ-5D, and for other variables of interest, were imputed using multiple imputation by chained equations (MICE), [28] incorporating the set of variables: age and sex; proxy-EQ-5D, NPI, Barthel ADL score, number of medical conditions—at baseline and follow-up; DEMQOL at follow up; as well as primary, inpatient, day-case, and outpatient care (trial and one year pre-admission) costs, social care (trial) costs, and permanent care home residence at baseline. To avoid bias, all variables included in the models for adjusted costs and QALYs in cost-effectiveness analysis were incorporated in the imputation. [28,29] In particular, since we imputed missing values of the models covariates, model outcomes (costs and follow-up EQ-5D determining QALYs) were included in the imputation model as well. [28,30] Additionally, by having the predictors of missing values for EQ-5D (and for other health status variables) in the imputation model (age, number of medical conditions, and care home residence at baseline) potential bias was reduced (MAR assumption was more plausible) and the standard errors in the adjustment multiply imputed models were minimised. [28]

One hundred imputed datasets were generated; based on the rule of thumb that the number of imputations was higher than the percentage of patients with at least one variable in the imputation model missing, equal to 94% [28] (percentages of missing values are at baseline and follow up, respectively: 33% and 56% (proxy EQ-5D), 1% and 15% (Barthel ADL), 53% and 25% (NPI), and 41% (DEMQOL, follow-up collected only)).

An alternative approach to deal with missing data, complete-case cost-effectiveness analysis, was applied. That is, 209/599 (34.9%) patients with complete QALY and trial cost data (210 patients), for whom one-year pre-admission secondary care cost data were also complete, were included in a complete-case cost-effectiveness analysis. In this approach, one year pre-admission secondary care cost and other covariates with complete data in the sub-sample of 209 patients were considered for the models for adjusted cost and QALYs. Due to the choice of adjustment covariates being the predictors of missing EQ-5D data at follow up, the MAR assumption was also sufficient to reduce bias in cost-effectiveness estimates. Hence, the unadjusted estimates were provided under missing completely at random (MCAR) assumption, while the MAR assumption was sufficient to justify complete-case adjusted cost-effectiveness analysis (CEA).

#### Full-sample (using imputed values) cost-effectiveness analysis

In the full-sample CEA, imputed missing primary care and EQ-5D data were incorporated into the generation of incremental costs and QALYs. Unadjusted costs in MMHU and standard care, as well as incremental costs by services were analysed, handling uncertainty by non-parametric bootstrapping. [31]

In the adjusted CEA, other variables with imputed missing values (one year pre-admission primary care cost, NPI and Barthel ADL score) were also used to adjust cost and QALYs for baseline characteristics. Finally, adjusted cost was estimated controlling for age, sex, EQ-5D utility index and permanent care home residence at baseline, and one year pre-admission healthcare cost. Adjusted QALYs were estimated controlling for age, sex, and baseline EQ-5D utility index, permanent care home residence, number of medical conditions, NPI, and Barthel ADL score. The adjustment models for both cost and QALYs included age, sex, EQ-5D index and permanent care home residence at baseline, as the explanatory variables which were predicted *a priori* to be the possible factors affecting both resource use and QALYs in the trial follow-up. Moreover, QALYs were controlled for baseline EQ-5D index as recommended for trial-based cost-utility analysis, [32] age and care home residence were found to be the predictors of missing QALYs values (see Table B-C in S4 Appendix), and block randomization was stratified for previous residence in a care home, which justified inclusion of these covariates in the adjustment models. Additionally, one year pre-admission healthcare cost was expected to be a strong predictor of trial resource use and costs. Baseline Barthel ADL, NPI, and number of medical conditions were included as the potential predictors of physical and mental health state at follow-up, and so QALYs were adjusted for these covariates. Baseline patient characteristics by trial arm, included in the adjusted CEA, are reported in Table A in S5 Appendix.

Regression techniques, employing a generalised linear model (GLM), were applied to adjust costs and QALYs by baseline characteristics. The appropriate distributional family type for the variance function was chosen by using the modified Park test; [33] Pregibon link and modified Hosmer-Lemeshow tests were used to diagnose any misspecification of the link function. [21] Regression models and diagnostic tests were calculated on each imputed dataset, to obtain adjusted cost and QALYs averaged across 100 imputations (Rubin’s rules), and to find the optimal GLMs for both costs and QALYs (considering the worst test results across imputations). Gamma distribution family and log link were chosen for costs, and normal family distribution and power link 0.25 were chosen for QALYs. [28] Adjusted patient cost and QALYs, calculated using the recycled prediction method, [21] were used to generate cost-effectiveness planes and cost-effectiveness acceptability curves (CEACs) on each imputed dataset; the full sample CEAC was obtained from the probability of cost-effectiveness for a given ceiling ratio, averaged across 100 imputations.

#### Complete-case cost-effectiveness analysis (alternative approach)

In the complete-case CEA, comprising 209/599 (34.9%) patients with complete QALY and trial cost data, for whom one year pre-admission secondary care cost data were also complete, unadjusted costs in MMHU and standard care were analysed, handling uncertainty by non-parametric bootstrapping. [31]

Adjusted cost was estimated controlling for age, sex, utility and permanent care home residence at baseline, and one year pre-admission secondary and tertiary care cost (pre-admission primary care costs are omitted here). Adjusted QALYs were estimated controlling for age, sex, and baseline utility, permanent care home residence, number of medical conditions, delirium at admission (defined by a score of at least 18/46 on the delirium rating scale (DRS-R-98 [34])) and severe cognitive impairment (Mini-Mental State Examination (MMSE) [35], MMSE ≤ 9). The reasons for inclusion of these baseline characteristics in the adjustment models were similar to the full-sample cost-effectiveness analysis. However, only covariates with complete data in the sub-sample of 209 patients were considered for the models for adjusted cost and QALYs. In particular, one year pre-admission secondary and tertiary care cost, permanent care home residence, and number of medical conditions were included as covariates in the models. To control QALYs for mental health status at baseline, DRS and MMSE variables were used, for which data for 2 and 1 participants, respectively, were missing in the complete-case CEA sub-sample (patients with missing DRS and MMSE baseline values were assumed to have delirium and severe cognitive impairment at admission). Baseline patient characteristics by trial arm, included in the adjusted complete-case CEA, are reported in Table B in S5 Appendix.

A diagnostic process was used to find the optimal GLMs for both costs and QALYs (the same tests as for the full-sample analysis). Gamma distribution family and power link 0.95 were chosen for costs, normal distribution family and power link 0.6 were chosen for QALYs. Recycled prediction method to generate adjusted patient cost and QALY was applied.[21]

## Results

### Intervention cost

Per-bed-day MMHU additional cost was £25. In the full sample, mean length of stay on MMHU was 15 days (95% confidence interval [CI]: 13, 16), and the mean per-patient cost of delivering the intervention (mean per-patient MMHU additional cost) was £368 (95% CI: 334, 410)–calculations are presented in Table 1.

### Full-sample (using imputed data) cost-effectiveness analysis

**Fig 1:**
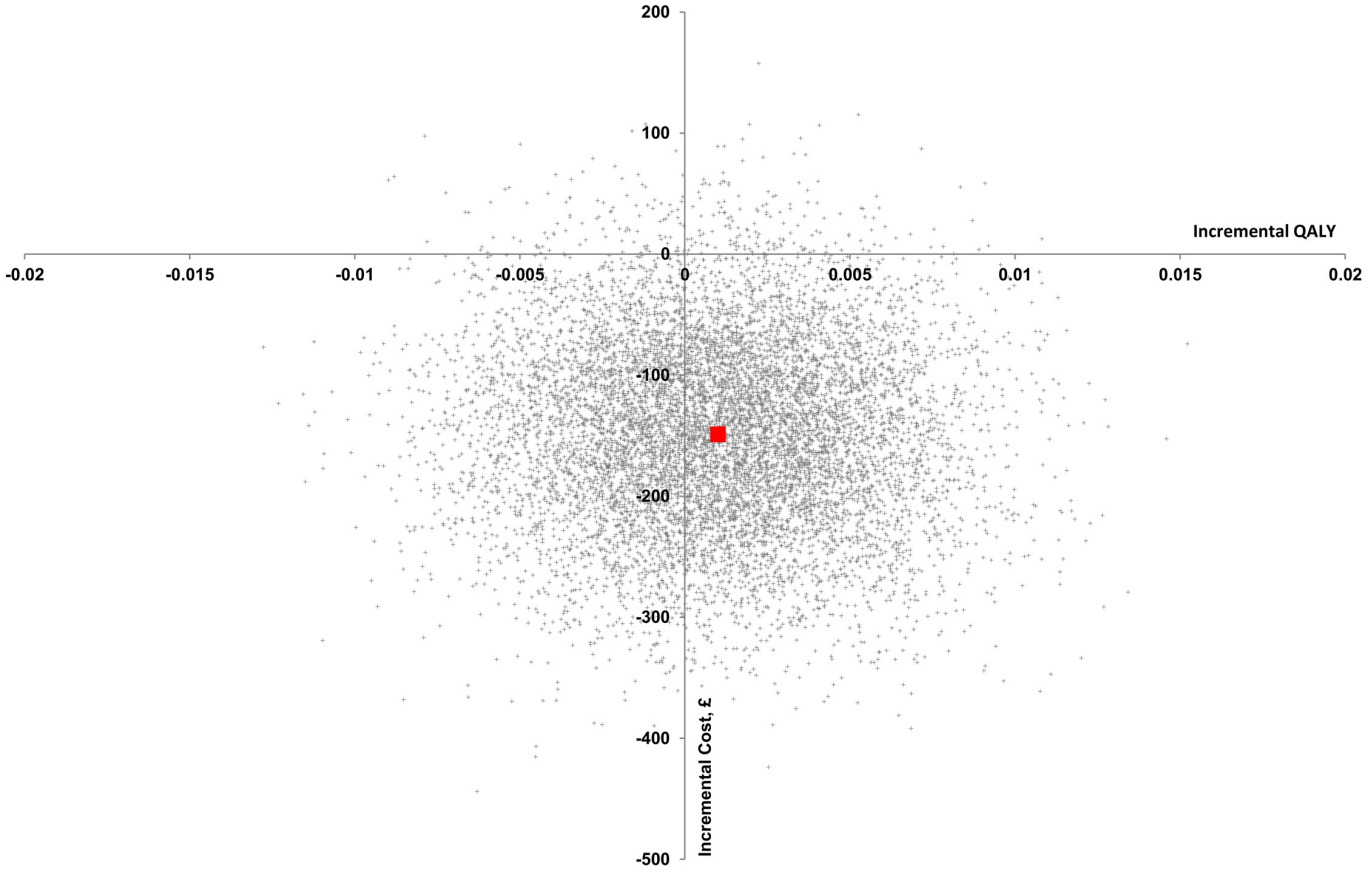
Cost-effectiveness plane–pairwise bootstrapping (adjusted analysis, full-sample imputed analysis). Bootstrapped incremental costs and QALYs were obtained for each imputation (5000 replications), and these were used in the full-sample cost-effectiveness analysis. Consequently, a cost-effectiveness plane should be drawn for 100 imputations (which would be impossible to present (100 ∙ 5000 = 500 000 points)). Hence, to approximate and illustrate the cost-effectiveness plane for the full-sample imputed analysis, 100 replications randomly chosen from each imputation were plotted in this figure (100 ∙ 100 = 10 000 points). The red square represents the point estimate: 0.001 QALY and -£149.

**Fig 2:**
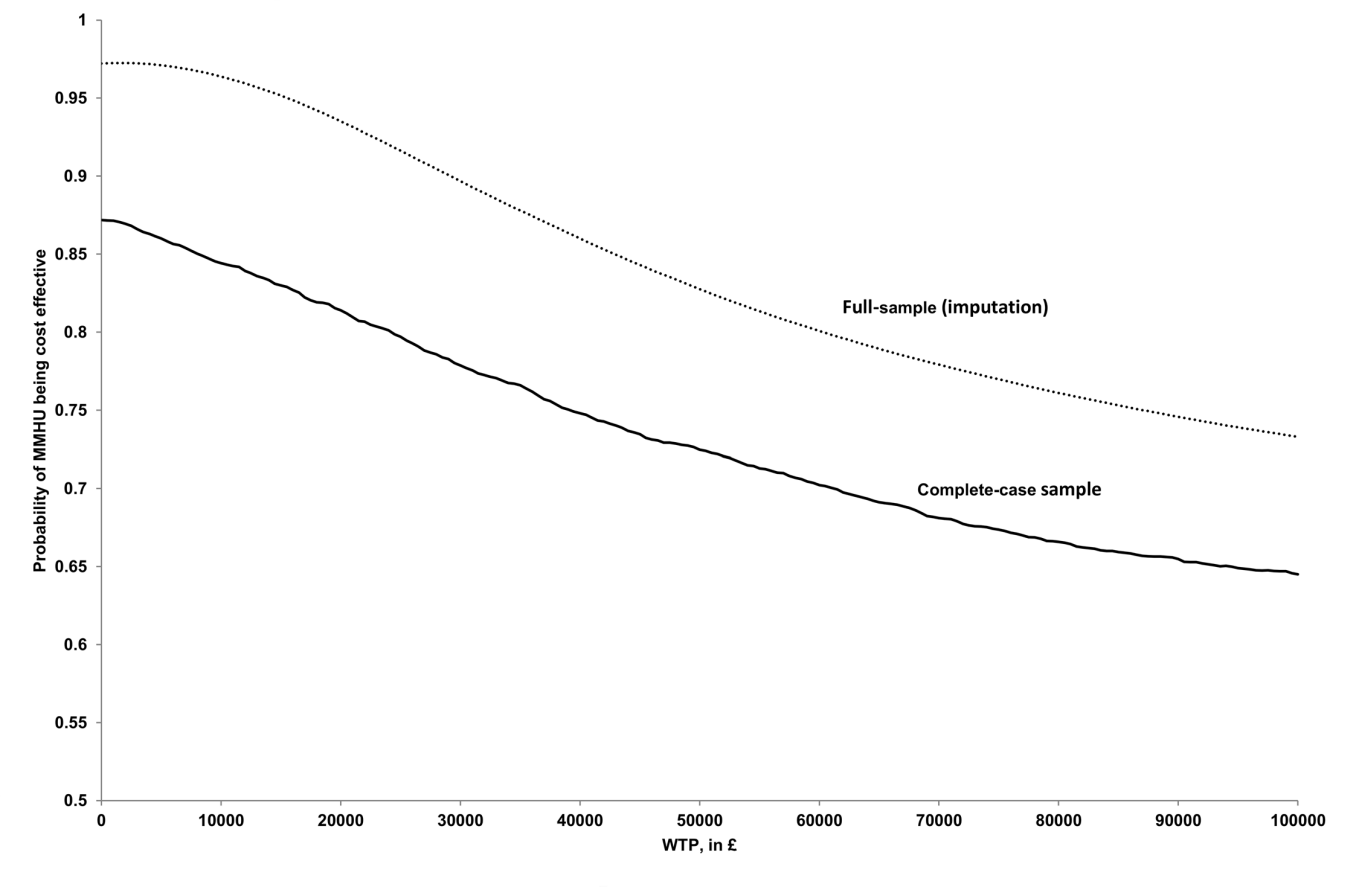
Cost-effectiveness acceptability curves (adjusted analyses)–full sample and complete-case analyses. Full-sample cost-effectiveness acceptability curve is obtained from probability of cost-effectiveness for given ceiling ratio, averaged across 100 imputations. CEACs represent probability of cost-effectiveness of MMHU for given WTP, where WTA is assumed to be equal to WTP (SW quadrant of cost-effectiveness plane, see Figs 1 and 3).

In the full-sample cost-effectiveness analysis (CEA), 599 (MMHU: 309) participants were analysed at 90-day follow-up, at which point 139 (MMHU: 68) were dead. In the unadjusted analysis, comparing the MMHU to standard care, the cost of inpatient care was non-significantly lower (-£434, 95% CI: -1199, 357), social care cost was non-significantly lower (-£194, 95% CI: -657, 301), and the cost of care (primary, secondary, tertiary and social care) was non-significantly lower (-£690, 95% CI: -1571, 246), resulting in incremental total cost of -£322 (95% CI: -1219, 621). The difference in QALYs gained was non-significant (0.008, 95% CI: -0.005, 0.020). In the adjusted CEA, the total cost for the MMHU was lower by -£149 (95% CI: -298, 4), with QALYs gained difference equal to 0.001 (95% CI: -0.006, 0.008), and a 58% probability of the MMHU being dominant (cost-saving with QALY benefit) and a 94% probability of cost-effectiveness (at a £20,000/QALY threshold). The probability of the MMHU being cost-saving with QALY loss (SW quadrant) was 39% (Tables 2 and 3, Figs 1 and 2).

**Table 2:** Full-sample cost-effectiveness analysis (mean cost in £ / mean QALYs, 95% CI).

	MMHU (309 patients)	Standard care^a^(290 patients)	Incremental cost / QALYs gained
**The cost of care** ^b^ ^c^	**7266 (6707, 7861)**	**7956 (7307, 8681)**	**-690 (-1571, 246)**
The cost of care–adjusted^d^	7345 (7248, 7441)	7862 (7758, 7965)	-517 (-660, -374)
Additional MMHU cost	368 (334, 410)	0	368 (334, 410)
***Total cost(care cost + MMHU cost)***	**7634 (7062, 8253)**	**7956 (7307, 8681)**	**-322 (-1219, 621)**
***Total cost- adjusted (care cost adjusted+ MMHU cost)***	***7714 (7606*, *7822)***	***7862 (7758*, *7965)***	***-149 (-298*, *4)***
**QALYs gained** ^b^	**0.111 (0.101, 0.121)**	**0.103 (0.093, 0.114)**	**0.008 (-0.005, 0.020)**
***QALYs gained–adjusted*** ^e^	***0*.*109 (0*.*102*, *0*.*116)***	***0*.*108 (0*.*101*, *0*.*114)***	***0*.*001 (-0*.*006*, *0*.*008)***
**ICER**			**MMHU dominant**
***ICER adjusted***			***MMHU dominant***

^a^Geriatric ward (204 patients) and general ward (86 patients).

^b^Primary care cost and QALY imputed using Multiple imputation by chained equation (MICE). Multiple imputation model applying predictive mean matching (pmm) for costs and utilities, and ordered logit (ologit) for Barthel ADL scores, DEMQOL, and NPI; 100 imputations generated.

^c^Healthcare (inpatient, day-case, outpatient, EMAS, MHT, critical care, primary care) and social care cost.

^d^Adjusted by age, sex, utility and permanent care home residence at baseline, and one year pre-admission healthcare cost care cost. A GLM model (family–gamma, link–log) was applied, as it was found to be optimal upon diagnostic procedure on each imputation (the worst test results across imputations were: Park test for gamma family, p-value = 0.05, Pregibon link test, p-value = 0.36, Hosmer-Lemeshow test, p-value = 0.11).

^e^Adjusted by age, sex, and baseline utility, permanent care home residence, number of medical conditions, NPI, and Barthel ADL. A GLM model (family–normal, link–power 0.25) was applied, as it was found to be optimal upon diagnostic procedure on each imputation (the worst test results across imputations were: Park test for normal family, p-value = 0.02, Pregibon link test, p-value = 0.50, Hosmer-Lemeshow test, p-value = 0.07, with Park test p-value being higher than 0.05 for 95% imputations and with average Park test p-value across imputations equal to 0.41).

**Table 3:** Full-sample cost analysis (mean cost in £, 95% CI).

	MMHU (309 patients)	Standard care^a^(290 patients)	Incremental cost / QALYs gained
Inpatient cost	5185 (4715, 5741)	5619 (5053, 6222)	-434 (-1199, 357)
Day-case cost	17 (6, 32)	60 (37, 93)	-42 (-77, -15)
Outpatient cost	174 (151, 199)	192 (169, 223)	-19 (-57, 16)
Primary care cost^b^	221 (200, 247)	206 (184, 232)	16 (-21, 47)
Critical care	8 (0, 22)	56 (2, 202)	-48 (-185, 10)
Ambulance service (EMAS)	26 (12, 44)	17 (8, 29)	9 (-9, 31)
Mental Health Trust (MHT)	110 (82, 141)	87 (59, 1276)	22 (-25, 65)
**Total healthcare cost**	**5741 (5261, 6298)**	**6238 (5648, 6908)**	**-496 (-1285, 320)**
**Social care cost**	**1525 (1236, 1830)**	**1718 (1363, 2126)**	**-194 (-657, 301)**
**The cost of care** ^c^	**7266 (6707, 7861)**	**7956 (7307, 8681)**	**-690 (-1571, 246)**

NB: the cost of the intervention is not included in these cost estimates. The cost of the intervention is presented in Table 2.

^a^Geriatric ward (204 patients) and general ward (86 patients).

^b^Primary care cost imputed using Multiple imputation by chained equation (MICE). Multiple imputation model applying predictive mean matching (pmm); 100 imputations generated.

**Fig 3:**
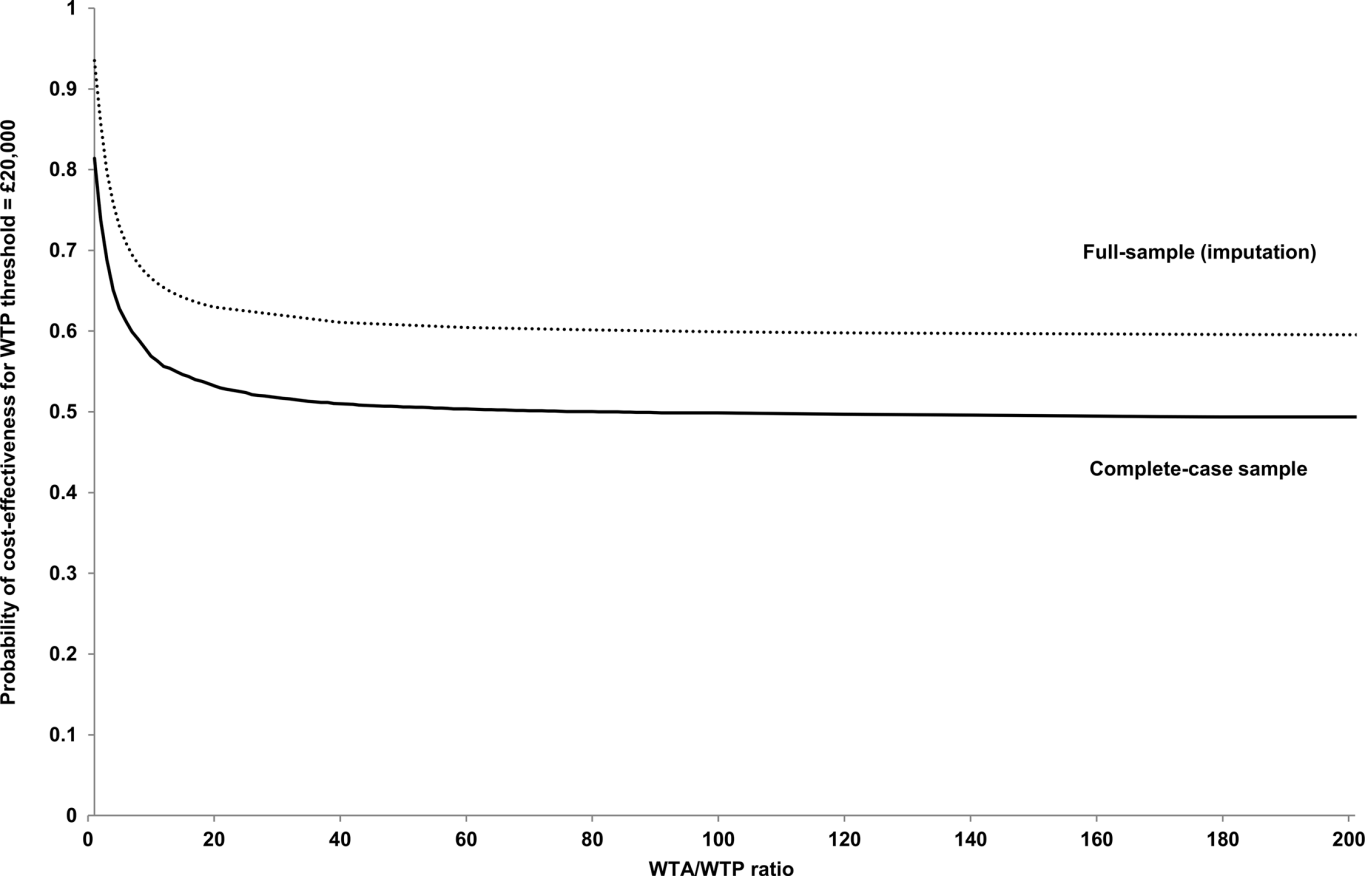
Probability of cost-effectiveness for WTP threshold equal to £20,000 in relation to WTA/WTP ratio–full sample and complete-case analyses.

Probability of cost-effectiveness for £20,000 WTP threshold in relation to WTA/WTP ratio is presented in Fig 3 (full-sample). It is shown that this probability goes down from 94% (WTA/WTP ratio equal to 1, as assumed in Fig 2 for the full-sample CEAC) to 86% for the ratio equal to 2, to 73% for the ratio equal to 5, approaching 59% for the infinite ratio (infinite WTA threshold—interpreted as non-acceptance of QALY loss for any amount of money saved).

### Complete-case cost-effectiveness analysis

**Fig 4:**
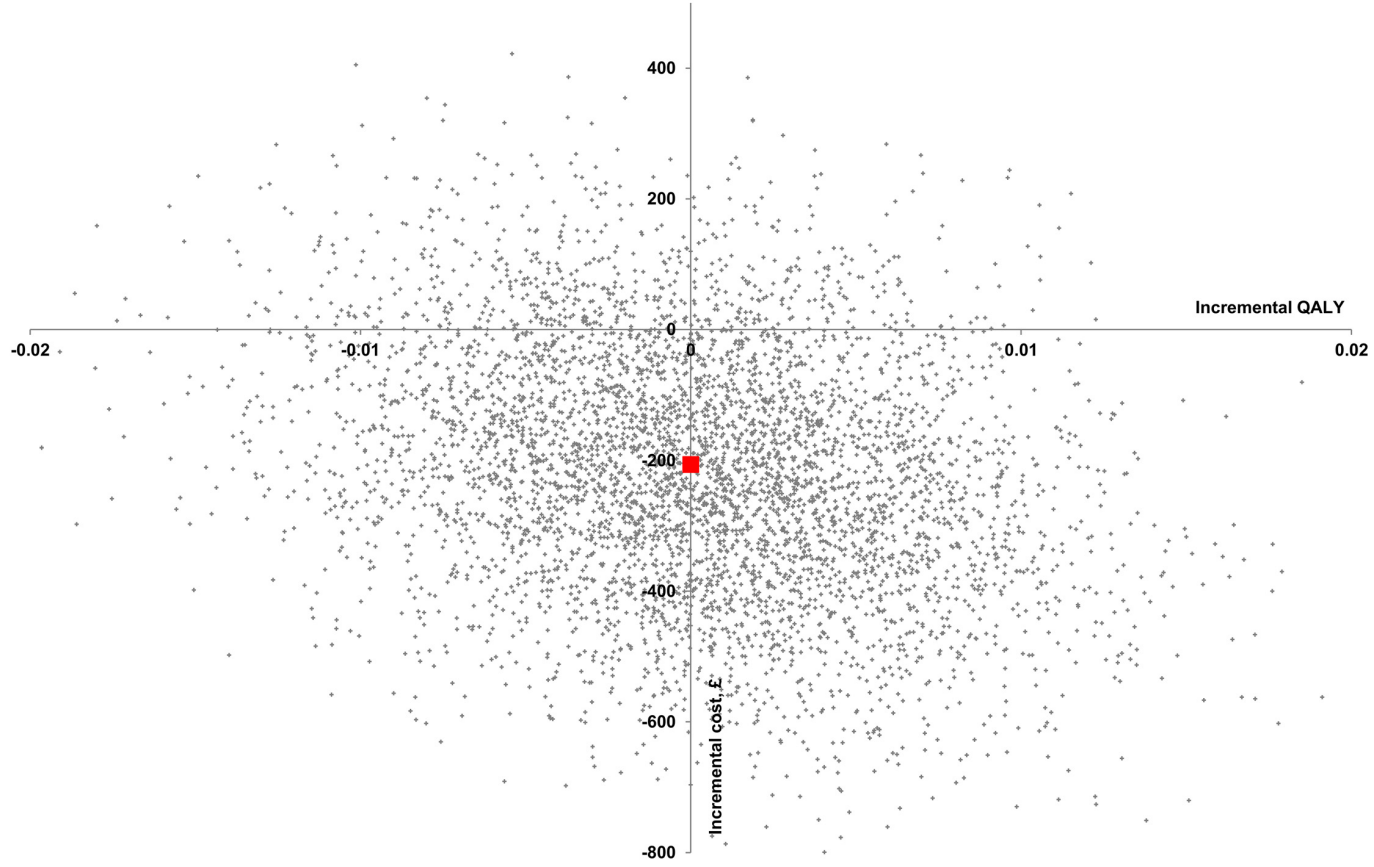
Cost-effectiveness plane–pairwise bootstrapping (adjusted analysis, complete case analysis). Red square represents point estimate 0.000 QALY and -£206.

In the subgroup of 209 (MMHU: 109) patients with complete QALY and resource-use data, including 49 (MMHU: 24) patients dead at follow up, comparing MMHU to standard care, the total cost was non-significantly lower (-£402, 95% CI: -2227, 1297) and the difference in QALYs gained was non-significant (0.007, 95% CI: -0.013, 0.027). In the adjusted CEA, the total cost for MMHU was lower (-£206, 95% CI: -591, 153) with no QALYs gained difference (0.000, 95% CI: -0.011, 0.011) and a 47% probability of the MMHU being dominant, and a 81% probability of cost-effectiveness (at a £20,000/QALY threshold). The probability of the MMHU being cost-saving with QALY loss (SW quadrant) was 40%. (Table 4, Figs 2 and 4)

**Table 4:** Complete-case cost-effectiveness analysis (mean cost in £ / mean QALYs, 95% CI).

	MMHU(109 patients)	Standard care^a^(100 patients)	Incremental cost / QALYs gained
The cost of care^b^	7430 (6399, 8631)	8203 (7052, 9751)	-772 (-2440, 942)
The cost of care–adjusted^c^	7553 (7311, 7807)	8130 (7888, 8385)	-577 (-833, -335)
Additional MMHU cost	371 (309, 440)	0	371 (309, 440)
**Total cost(care cost + MMHU cost)**	**7801 (6720, 9031)**	**8203 (7052, 9751)**	**-402 (-2227, 1297)**
***Total cost—adjusted (care cost adjusted + MMHU cost)***	***7924 (7654*, *8197)***	***8130 (7888*, *8385)***	***-206 (-591*, *153)***
**QALYs gained**	**0.123 (0.109, 0.137)**	**0.116 (0.102, 0.130)**	**0.007 (-0.013, 0.027)**
***QALYs gained–adjusted*** ^d^	***0*.*120 (0*.*112*, *0*.*128)***	***0*.*120 (0*.*112*, *0*.*127)***	***0*.*000 (-0*.*011*, *0*.*011)***
**ICER**			**MMHU dominant**
***ICER adjusted***			***MMHU dominant***

^a^Geriatric ward (66 patients) and general ward (34 patients).

^b^Inpatient, day-case, ambulance service (EMAS), Mental Health Trust (MHT), critical care, outpatient, primary care, and social care.

^c^Adjusted by age, sex, utility and permanent care home residence at baseline, and one year pre-admission secondary care cost. A GLM model (family—gamma, power link—0.95) was applied. Park test for gamma family, p-value = 0.92, Pregibon link test, p-value = 0.39, Hosmer-Lemeshow test, p-value = 0.36.

^d^Adjusted by age, sex, and baseline utility, permanent care home residence, number of medical conditions, delirium (DRS-R-98 > 17.75) and severe cognitive impairment (MMSE ≤ 9). A GLM model (family—normal, power link—0.6) was applied. Park test for normal family, p value = 0.07, Pregibon link test, p-value = 0.68, Hosmer-Lemeshow test, p-value = 0.20.

Probability of cost-effectiveness for £20,000 WTP threshold in relation to WTA/WTP ratio is presented in Fig 3 (complete-case). This probability goes down from 81% (WTA/WTP ratio equal to 1, as assumed in Fig 2 for the complete-case CEAC) to 74% for ratio equal to 2, to 63% for ratio equal to 5, approaching 48% for infinite ratio (infinite WTA threshold). (Fig 3)

## Discussion

### Summary of results

The specialist unit for people with delirium and dementia did not demonstrate convincing benefits in health status over usual hospital care, as no significant effect on QALY gain was observed. However, the results did show a trend towards cost savings and a high probability of cost-effectiveness (94%) from a combined health and social care perspective, when usual criteria were applied. When excluding the cases in which there were cost savings but worse outcomes (QALY loss), the probability of cost-effectiveness fell to 59%.

### Internal validity

The strengths of this study were that it was conducted as part of a RCT rather than a less robust design, resource-use ascertainment was by extraction from electronic datasets rather than recall enhancing the quality of the data and hence results, and multiple resource-use datasets were examined to produce a more comprehensive estimate of costs than using a single and potentially unreliable data source. The economic evaluation was conducted independently of the clinical service and, in large part, independently of the investigators who had designed and implemented the clinical effectiveness evaluation.

There were considerable missing data, due to the inability of frail and cognitively impaired participants to complete EQ-5D, and a systematic difference in values for proxy compared with self-completed EQ-5D. Hence imputation was used, incorporating proxy EQ-5D and other clinical measures, to estimate the true impact of MMHU care on patients’ health status, which could be a source of error. Employing an alternative approach omitting missing data (complete-case analysis) showed no major differences in results; however, we did not ascertain informal care or privately funded costs, meaning that our findings are limited to the health and social care service perspective. Informal care costs form an important part of total costs for people with dementia. [36] The findings represent a comparison between the MMHU and standard care. However, 70% of standard care was situated on specialist acute geriatric medical wards delivering comprehensive geriatric assessment, which is known to deliver better health outcomes than general internal medical wards for frail older people (that is, an ‘active control’). [37] The impact of the MMHU on health status may therefore have been understated compared with less specialist care.

The EQ-5D has limitations as a preference-based generic health status measure for calculating QALYs in frail and cognitively impaired older people with progressive conditions. Firstly, the EQ-5D is a simple, five dimension, 3-level measure of health status which may be insensitive to changes in health that are important in this context. [38,39] There is some evidence to support the EQ-5D as a valid measure for assessing quality of life in older people, [40] including people with cognitive impairment using proxies when necessary. [41] Due to the advocacy by NICE to use the EQ-5D for comparability between studies, and the lack of other, more sensitive preference-based measures which can be used to elicit the QALY, the EQ-5D was the best preferred option for performing this economic evaluation. At the time of planning this study the DEMQOL, a condition specific quality of life measure for use in older people with dementia, [42] did not have a valid preference-based scoring tariff. The DEMQOL may be more sensitive for measuring condition-specific quality of life but was no different when measured in survivors at the end of the follow up period. [8] More recently the UDEMQOL has been developed as a preference-based version of the DEMQOL which can be used as a condition-specific preference-based measure for eliciting the QALY. [43,44] The UDEMQOL can be used to provide complimentary results for comparison with the EQ-5D [13,44] and should be considered for use in future studies if further studies establish its validity in this setting. [44] Secondly, the QALY as elicited by the EQ-5D is a unidimensional metric of change in health status over years of life, and therefore does capture broader aspects of well-being, [45,46] capability [47–50] or the ‘spillover’ effect on carers [51,52] that may have been affected by the intervention. These aspects are increasingly recognised as areas that should be accounted for when assessing the economic outcome of trials. [13,48,52] Evidence from the TEAM trial showed that participants on the MMHU spent significantly more time with positive mood or engagement, and experienced more staff interactions that addressed emotional and psychological needs. [8] Additionally, more family carers in MMHU arm were satisfied with care. For these reasons, we believe our analysis only presents a partial assessment of the overall benefit of the intervention.

This economic evaluation was derived from trial data up to three months of follow-up, without measuring or modelling the health and cost outcomes beyond this horizon. However, given the fast moving changes in clinical conditions of patients, the health and cost effects of MMHU care are likely to be limited to a short period after hospital stay, and the trial follow-up was long enough to assess effects of the MMHU (cf. trial protocol [7] and [8]), although the trends towards cost savings (such as from long term care) may have been stronger if we had data from a longer period of follow up.

### External validity/context

This is the first study of this specific model of care: no cost-effectiveness analyses of specialist unit care for cognitively impaired frail older people have been identified. [53] However, the patient group involved and the core processes of the MMHU were similar to the patient groups and core processes involved in services delivering comprehensive geriatric assessment (CGA), where a potential cost reduction compared with general medical care has been observed. [53,54] Thus this study contributes towards, and is compatible with, a small evidence base about the economic consequences of CGA.

The economic impact of the health and social care of older people has been rarely described fully. [36,55] Despite recommendations to assess opportunity costs [56], only half of published studies measured costs other than secondary care, even fewer including long term or social care costs: the eight studies reporting costs in CGA trials in a recent review only reported costs from a hospital perspective, and so did not investigate whether costs were shifted to other areas of health care, or to social care or informal carers. [54] Thus, this study is an important contribution to the evidence base, particularly because around 1/3 of the cost savings observed in this study were non-hospital costs (social and primary care). Despite the fact that cost savings shown were only small percentages of the total care cost occurred in the standard care arm (4% and 2%, for unadjusted and adjusted costs, respectively), the potential cost savings for the NHS could be large if similar specialist dementia care is implemented in the UK hospitals.

### What the results mean

The value of these findings depends upon the degree to which the findings from economic studies based on trials that were not positive for their primary outcome are judged by those using such information, and the degree to which conventional cost-effectiveness estimates are judged when they rely considerably upon cases in which there were cost savings but QALY losses. Health care funders may find that cost-effectiveness findings based upon QALY gains at additional cost (willingness to pay, WTP) are more acceptable than cost savings and QALY losses (willingness to accept, WTA)–an issue discussed widely in the health and behavioral economics literature (cf. [24–26]). Hence, we provided the sensitivity analysis to incorporate possible WTA/WTP disparity, by estimating probability of cost-effectiveness dependent on the value of WTA/WTP ratio. [24] Due to unknown decision makers’ preference over WTA/WTP, the interpretation of such sensitivity analysis is limited to the extreme in which small QALY loss is not accepted for any level of cost-savings. In this study, the interpretation is even more difficult because of the possibility that the overall benefits of the intervention may have been understated in the economic analysis. However, we conclude that there are sufficient grounds for further development of evaluation of specialist medical and mental health units.

The further development and evaluation of this comprehensive model of care can be guided by the results of this study. For example, this study illustrates the potential value of determining a wider range of health and social costs to appraise the total impact of services. Given that considerable effort was put into discharge planning, communication with families and care homes, referral to community services, and advance care planning, it is likely that the accumulation of multiple small incremental improvements in multiple processes and outcomes can only be observed when multiple sources of costs across the health and social care system are taken into account.

Mortality was high in the population studied (25% at 90 days).[8] It is difficult to define measurable outcomes in studies of palliative and supportive care, but patient experience and carer satisfaction are likely to be important. The NHS Outcomes framework includes ‘a positive experience of care’ as one of its five domains. [57] Tools widely used to measure health care outcomes in economic evaluations do not appear to discriminate well in the end-of-life care context, [58] so carer preferences should be incorporated in healthcare decision making. [59] Economic evaluation of such services may need to consider broader outcomes than the QALY. For example, a recent study has shown the advantages of multiple domain comparisons, emphasizing transparency and better informing reimbursement and research decisions when using this approach. [60] Therefore, considering the totality of outcomes, including patient experience and carer satisfaction (a cost-consequences analysis), would emphasize effects that may be more appropriate for frail older patients, often approaching the end of life. An alternative would be a cost minimization approach. Our findings suggest that care on the specialist unit was preferable (better quality and experience even if health status was no different). In this case, costs can be compared to determine preference. In this study, we showed a trend towards cost reduction in the MMHU arm, and hence a trend towards superiority.

In conclusion, further development and evaluation of specialist units in general hospitals for people with dementia and delirium is warranted based on the fact that the unit studied here led to better quality of care, [8] has a reasonable probability of cost-effectiveness even when cost saving QALY losing cases are not included in the estimate of cost-effectiveness, and showed a trend towards cost-savings when a cost minimisation approach is taken. Such units should be seen as an important response to the challenge of managing mental health conditions in general hospitals, in addition to liaison old age psychiatry services. Further research of similar services should aim to find better ways of capturing health benefits in patient groups receiving palliative and supportive care, and use multiple cost sources to assess the full cost impact.

## Supporting Information

S1 Checklist CONSORT checklist for TEAM clinical trial manuscript. Goldberg SE, Bradshaw LE, Kearney FC, et al. Care in specialist medical and mental health unit compared with standard care for older people with cognitive impairment admitted to general hospital: randomised controlled trial (NIHR TEAM trial). BMJ 2013;**347** doi: 10.1136/bmj.f4132[.
(DOC)

S1 Appendix CHEERS Statement for the TEAM economic evaluation study. (DOCX)

S2 Appendix Summary of resource-use parameters obtained in the TEAM trial. (DOCX)

S3 Appendix Description and breakdown of HRG codes used in costing of hospital admission data. (DOCX)

S4 Appendix Missing data patterns and predictors. Proportions missing between groups for variables of interest **(Table A)**. Logistic regression: predictors of missing value for baseline EQ-5D, 599 observations **(Table B)**. Logistic regression: predictors of missing value for follow-up EQ-5D,460 observations **(Table C)**. Logistic regression: predictors of missing value for follow-up proxy EQ-5D, 460 observations **(Table D)**. Logistic regression: predictors of missing value for follow-up Barthel ADL, 460 observations **(Table E)**. Logistic regression: predictors of missing value for follow-up DEMQOL, 460 observations **(Table F)**.
(DOCX)

S5 Appendix Baseline characteristics by trial arm. Baseline characteristics–covariates included in the full-sample adjusted CEA **(Table A)**. Baseline characteristics–covariates included in the complete-case adjusted CEA **(Table B)**.
(DOCX)

S1 Protocol Harwood R, Goldberg S, Whittamore K, et al. Evaluation of a Medical and Mental Health Unit compared with standard care for older people whose emergency admission to an acute general hospital is complicated by concurrent 'confusion': a controlled clinical trial. Acronym: TEAM: Trial of an Elderly Acute care Medical and mental health unit. Trials 2011;**12**(1):123.
(PDF)

## References

[1] Boustani M, Baker M, Campbell N, Munger S, Hui S, Castelluccio P, et al (2010) Impact and recognition of cognitive impairment among hospitalized elders. J Hosp Med 5 (2): 69–75. 10.1002/jhm.589 20104623 PMC2814975

[2] Goldberg S, Whittamore K, Harwood R, Bradshaw L, Gladman J, Jones R (2012) The prevalence of mental health problems amongst older adults admitted as an emergency to a general hospital. Age Ageing 41: 80–86. 10.1093/ageing/afr106 21890483 PMC3234074

[3] Royal College of Psychiatrists (2005) *Who Cares Wins* *Improving the outcome for older people admitted to the general hospital* *Guidelines for the development of Liaison Mental Health Services for older people*.

[4] Department of Health (2009) Living Well With Dementia: A National Dementia Strategy.

[5] Holmes J, Montaňa C, Powell G, Hewison J, House A, Mason J, et al. (2010) Liaison Mental Health Services for Older People: A Literature review, service mapping and in-depth evaluation of service models.

[6] Harwood RH, Porock D, King N, Edwards G, Hammond S, Howe L, et al (2010) Development of a specialist medical and mental health unit for older people in an acute general hospital. Medical Crises in Older People Discussion paper series.

[7] Harwood R, Goldberg S, Whittamore K, Russell C, Gladman J, Jones R, et al (2011) Evaluation of a Medical and Mental Health Unit compared with standard care for older people whose emergency admission to an acute general hospital is complicated by concurrent 'confusion': a controlled clinical trial. Acronym: TEAM: Trial of an Elderly Acute care Medical and mental health unit. Trials 12: 123 10.1186/1745-6215-12-123 21569471 PMC3117715

[8] Goldberg SE, Bradshaw LE, Kearney FC, Russell C, Whittamore KH, Foster PER, et al (2013) Care in specialist medical and mental health unit compared with standard care for older people with cognitive impairment admitted to general hospital: randomised controlled trial (NIHR TEAM trial). BMJ 347. 10.1136/bmj.f4132 PMC3698942 23819964

[9] Welsh TJ, Gordon AL, Gladman JR (2014) Comprehensive geriatric assessment–a guide for the non-specialist. International Journal of Clinical Practice 68: 290–293. 10.1111/ijcp.12313 24118661 PMC4282277

[10] Sampson EL, Blanchard MR, Jones L, Tookman A, King M (2009) Dementia in the acute hospital: prospective cohort study of prevalence and mortality. Br J Psychiatry 195: 61–66. 10.1192/bjp.bp.108.055335 19567898

[11] EuroQol Group:EuroQol (1990) A new facility for the measurement of health related quality of life. Health Policy 16: 199–208. 10109801 10.1016/0168-8510(90)90421-9

[12] Dolan P (1997) Modeling valuations for EuroQol health states. Med Care 35: 1095–1108. 9366889 10.1097/00005650-199711000-00002

[13] National Institute for Clinical E (2013) Guide to the Methods of Technology Appraisal National Institute for Clinical Excellence London. 27905712

[14] Smith SC, Lamping DL, Banerjee S, Harwood R, Foley BP, Cook JC, et al (2005) Measurement of health related quality of life for people with dementia: development of a new instrument (DEMQOL) and an evaluation of current methodology. Health Technology Assessment (Winchester, England) 9: 1–93, iii-iv. 10.3310/hta9100 15774233

[15] Cummings JL, Mega M, Gray K, Rosenberg-Thompson S, Carusi DA, Gornbein J (1994) The Neuropsychiatric Inventory: comprehensive assessment of psychopathology in dementia. Neurology 44: 2308–2314. 7991117 10.1212/wnl.44.12.2308

[16] Wade DT, Collin CL (1988) The Barthel ADL index: a standard measure of physical disability? Int Dis Studies 10: 64–67. 10.3109/09638288809164105 3042746

[17] Curtis L (2012) Unit costs of health and social care 2012 University of Kent, Personal Social Services Research Unit.

[18] Franklin M, Berdunov V, Edmans J, Conroy S, Gladman J, Tanajewski L, et al (2014) Identifying patient-level health and social care costs for older adults discharged from acute medical units in England. Age and Ageing 43: 703–707. 10.1093/ageing/afu073 25059421

[19] National Audit Office (2011) The National Programme for IT in the NHS: an update on the delivery of detailed care records systems

[20] NHS Careers (2011) Agenda for change–pay rates.

[21] Glick HA, Doshi JA, Sonnad SS, Polsky D (2007) Economic Evaluation in Clinical Trials (Handbooks for Health Economic Evaluation); Oxford University Press, editor. Oxford.

[22] Fenwick E, Byford S (2005) A guide to cost-effectiveness acceptability curves. The British Journal of Psychiatry 187: 106–108. 16055820 10.1192/bjp.187.2.106

[23] Fenwick E, Claxton K, Sculpher MJ (2001) Representing uncertainty: the role of cost effectiveness acceptability curves. Health Economics 10: 779–787. 11747057 10.1002/hec.635

[24] Severens J, Brunenberg DM, Fenwick EL, O’Brien B, Joore M (2005) Cost-effectiveness acceptability curves and a reluctance to lose. PharmacoEconomics 23: 1207–1214. 16336015 10.2165/00019053-200523120-00005

[25] Dubourg WR, Jones-Lee MW, Loomes G (1994) Imprecise preferences and the WTP-WTA disparity. Journal of Risk and Uncertainty 9: 115–133.

[26] O'Brien BJ, Gertsen K, Willan AR, Faulkner A (2002) Is there a kink in consumers' threshold value for cost-effectiveness in health care? Health Economics 11: 175–180. 11921315 10.1002/hec.655

[27] StataCorp LP (2008) Stata data analysis and statistical Software. Special Edition Release 101 edition.

[28] White IR, Royston P, Wood AM (2011) Multiple imputation using chained equations: Issues and guidance for practice. Stat Med 30: 377–399. 10.1002/sim.4067 21225900

[29] Knorr-Held L (2000) Analysis of Incomplete Multivariate Data. Schafer J. L., Chapman & Hall, London, 1997. No. of pages: xiv+430. Price: £39.95. ISBN 0-412-04061-1. Statistics in Medicine 19: 1006–1008.

[30] Moons KGM, Donders RART, Stijnen T, Harrell FE Jr (2006) Using the outcome for imputation of missing predictor values was preferred. Journal of Clinical Epidemiology 59: 1092–1101. 16980150 10.1016/j.jclinepi.2006.01.009

[31] Briggs A, Gray A (1998) The distribution of health care costs and their statistical analysis for economic evaluation. J Health Serv Res Policy 3: 233–245. 10187204 10.1177/135581969800300410

[32] Manca A, Hawkins N, Sculpher MJ (2005) Estimating mean QALYs in trial-based cost-effectiveness analysis: the importance of controlling for baseline utility. Health Economics 14: 487–496. 15497198 10.1002/hec.944

[33] Manning WG, Mullahy J (2001) Estimating log models: to transform or not to transform? Journal of Health Economics 20: 461–494. 11469231 10.1016/s0167-6296(01)00086-8

[34] Trzepacz PT, Mittal D, Torres R, Kanary K, Norton J, Jimerson N (2001) Validation of the Delirium Rating Scale-revised-98: comparison with the delirium rating scale and the cognitive test for delirium. J Neuropsychiatry Clin Neurosci 13: 229–242. 11449030 10.1176/jnp.13.2.229

[35] Folstein MF, Folstein SE, McHugh PR (1975) ‘Mini-mental state’. A practical method for grading the cognitive state of patients for the clinician. Journal of Psychiatric Research 12: 189–198. 1202204 10.1016/0022-3956(75)90026-6

[36] SCHNEIDER J, HALLAM A, ISLAM MK, MURRAY J, FOLEY B, ATKINS L, et al (2003) Formal and informal care for people with dementia: variations in costs over time. Ageing & Society 23: 303–326.

[37] Ellis G, Whitehead MA, Robinson D, O’Neill D, Langhorne P (2011) Comprehensive geriatric assessment for older adults admitted to hospital: meta-analysis of randomised controlled trials. 10.1136/bmj.d6553 PMC3203013 22034146

[38] Brazier JE, Walters SJ, Nicholl JP, Kohler B (1996) Using the SF-36 and Euroqol on an elderly population. Quality of Life Research 5: 195–204. 8998488 10.1007/BF00434741

[39] Holland R, Smith RD, Harvey I, Swift L, Lenaghan E (2004) Assessing quality of life in the elderly: a direct comparison of the EQ-5D and AQoL. Health Economics 13: 793–805. 15322991 10.1002/hec.858

[40] Haywood KL, Garratt AM, Fitzpatrick R (2005) Quality of life in older people: A structured review of generic self-assessed health instruments. Quality of Life Research 14: 1651–1668. 16119178 10.1007/s11136-005-1743-0

[41] Wolfs CAG, Dirksen CD, Kessels A, Willems DCM, Verhey FRJ, Severens JL (2007) Performance of the EQ-5D and the EQ-5D+C in elderly patients with cognitive impairments. Health and Quality of Life Outcomes 5: 33–33. 17570832 10.1186/1477-7525-5-33 PMC1904437

[42] Smith SC, Lamping DL, Banerjee S, Harwood R, Foley B, Smith P, et al (2005) Measurement of health-related quality of life for people with dementia: development of a new instrument (DEMQOL) and an evaluation of current methodology. Health Technol Assess 9: 1–93, iii-iv. 10.3310/hta9100 15774233

[43] Mulhern B, Smith SC, Rowen D, Brazier JE, Knapp M, Lamping DL, et al Improving the Measurement of QALYs in Dementia: Developing Patient- and Carer-Reported Health State Classification Systems Using Rasch Analysis. Value in Health 15: 323–333. 10.1016/j.jval.2011.09.006 22433764

[44] Mulhern B, Rowen D, Brazier J, Smith S, Romeo R, Tait R, et al (2013) Development of DEMQOL-U and DEMQOL-PROXY-U: generation of preference-based indices from DEMQOL and DEMQOL-PROXY for use in economic evaluation. Health Technol Assess 17: v–xv, 1–140. 10.3310/hta17050 PMC4781552 23402232

[45] Cooper R, Stafford M, Hardy R, Aihie Sayer A, Ben-Shlomo Y, Cooper C, et al (2014) Physical capability and subsequent positive mental wellbeing in older people: findings from five HALCyon cohorts. Age 36: 445–456. 10.1007/s11357-013-9553-8 23818103 PMC3818137

[46] Tennant R, Hiller L, Fishwick R, Platt S, Joseph S, Weich S, et al (2007) The Warwick-Edinburgh Mental Well-being Scale (WEMWBS): development and UK validation. Health and Quality of Life Outcomes 5: 63–63. 18042300 10.1186/1477-7525-5-63 PMC2222612

[47] Coast J, Smith RD, Lorgelly P (2008) Welfarism, extra-welfarism and capability: The spread of ideas in health economics. Social Science & Medicine 67: 1190–1198. 18657346 10.1016/j.socscimed.2008.06.027

[48] Coast J, Flynn TN, Natarajan L, Sproston K, Lewis J, Louviere JJ, et al (2008) Valuing the ICECAP capability index for older people. Social Science & Medicine 67: 874–882. 18572295 10.1016/j.socscimed.2008.05.015

[49] Coast J, Peters T, Natarajan L, Sproston K, Flynn T (2008) An assessment of the construct validity of the descriptive system for the ICECAP capability measure for older people. Quality of Life Research 17: 967–976. 10.1007/s11136-008-9372-z 18622721

[50] Al-Janabi H, N Flynn T, Coast J (2012) Development of a self-report measure of capability wellbeing for adults: the ICECAP-A. Quality of Life Research 21: 167–176. 10.1007/s11136-011-9927-2 21598064 PMC3254872

[51] Al-Janabi H, Flynn T, Coast J (2011) QALYs and Carers. PharmacoEconomics 29: 1015–1023. 10.2165/11593940-000000000-00000 22077576

[52] Al-Janabi H, Flynn TN, Coast J (2011) Estimation of a preference-based carer experience scale. Medical Decision Making 31: 458–468. 10.1177/0272989X10381280 20924044

[53] George J, Adamson J, Woodford H (2011) Joint geriatric and psychiatric wards: a review of the literature. Age Ageing 40: 543–548. 10.1093/ageing/afr080 21784760

[54] Ellis G, Whitehead MA, Robinson D, O’Neill D, Langhorne P (2011) Comprehensive geriatric assessment for older adults admitted to hospital: meta-analysis of randomised controlled trials. BMJ 343. 10.1136/bmj.d6553 PMC3203013 22034146

[55] Mason A, Weatherly H, Spilsbury K, Arksey H, Golder S, Adamson J, et al (2007) A systematic review of the effectiveness and cost-effectiveness of different models of community-based respite care for frail older people and their carers. Health Technology Assessment 11: 1–157, iii. 10.3310/hta11150 17459263

[56] National Institute for Clinical Excellence (2004) Guide to the methods of technology appraisal. 27905712

[57] Department of H (2013) The NHS outcomes framework 2014/15 Department of Health London.

[58] NORMAND C (2012) Setting priorities in and for end-of-life care: challenges in the application of economic evaluation. Health Economics, Policy and Law 7: 431–439. 10.1017/S1744133112000229 23079301

[59] Al-Janabi H, McCaffrey N, Ratcliffe J (2013) Carer Preferences in Economic Evaluation and Healthcare Decision Making. The Patient—Patient-Centered Outcomes Research 6: 235–239. 10.1007/s40271-013-0035-y 24190630

[60] McCaffrey N, Agar M, Harlum J, Karnon J, Currow D, Eckermann S (2015) Better Informing Decision Making with Multiple Outcomes Cost-Effectiveness Analysis under Uncertainty in Cost-Disutility Space. PLoS ONE 10: e0115544 10.1371/journal.pone.0115544 25751629 PMC4353730

