# Supplementary material for: Economic Evaluation of a General Hospital Unit for Older People with Delirium and Dementia (TEAM Randomised Controlled Trial)
Source: PLoS One. 2015 Dec 18;10(12):e0140662. doi: 10.1371/journal.pone.0140662 (PMC4687694; doi:10.1371/journal.pone.0140662)
Supplement: S1 Checklist — Goldberg SE, Bradshaw LE, Kearney FC, et al. Care in specialist medical and mental health unit compared with standard care for older people with cognitive impairment admitted to general hospital: randomised controlled trial (NIHR TEAM trial). BMJ 2013;347 doi: 10.1136/bmj.f4132[. (DOC) [file pone.0140662.s001.doc]

**Checklist S1: CONSORT statement for TEAM trial [Goldberg SE et. al. *BMJ* 2013 [8]]**

**Checklist of Items for Reporting Trials of Nonpharmacologic Treatments***

| **Section** | **Item** | **Standard CONSORT Description** | **Extension for Nonpharmacologic Trials** | **Reported on Page No.** |
| --- | --- | --- | --- | --- |
| Title and abstract† | 1 | How participants were allocated to interventions (e.g., “random allocation,” “randomized,” or “randomly assigned”) | In the abstract, description of the experimental treatment, comparator, care providers, centers, and blinding status | Page 1 and 2 |
| **Introduction** |  |  |  |  |
| Background | 2 | Scientific background and explanation of rationale |  | Page 3 |
| **Methods** |  |  |  |  |
| Participants† | 3 | Eligibility criteria for participants and the settings and locations where the data were collected | When applicable, eligibility criteria for centers and those performing the interventions | Page 4 para 1  Page 3 para 3 |
| Interventions† | 4 | Precise details of the interventions intended for each group and how and when they were actually administered | Precise details of both the experimental treatment and comparator | Page 4 para 3  Page 5 |
|  | 4A |  | Description of the different components of the interventions and, when applicable, descriptions of the procedure for tailoring the interventions to individual participants | Page 5 para 1 |
|  | 4B |  | Details of how the interventions were standardized | n/a |
|  | 4C |  | Details of how adherence of care providers with the protocol was assessed or enhanced | Page 7 para 1 |
| Objectives | 5 | Specific objectives and hypotheses |  | Page 3 para 2 |
| Outcomes | 6 | Clearly defined primary and secondary outcome measures and, when applicable, any methods used to enhance the quality of measurements (e.g., multiple observations, training of assessors) |  | Page 5 para 3&4 |
| Sample size† | 7 | How sample size was determined and, when applicable, explanation of any interim analyses and stopping rules | When applicable, details of whether and how the clustering by care providers or centers was addressed | Page 8 para 3 |
| Randomization–  sequence generation† | 8 | Method used to generate the random allocation sequence, including details of any restriction (e.g., blocking, stratification) | When applicable, how care providers were allocated to each trial group | Page 4 para 2 |
| Allocation concealment | 9 | Method used to implement the random allocation sequence (e.g., numbered containers or central telephone), clarifying whether the sequence was concealed until interventions were assigned |  | Page 4 para 2 |
| Implementation | 10 | Who generated the allocation sequence, who enrolled participants, and who assigned participants to their groups |  | Page 4 para 2 |
| Blinding (masking)† | 11A | Whether or not participants, those administering the interventions, and those assessing the outcomes were blinded to group assignment | Whether or not those administering co-interventions were blinded to group assignment | Page 4 para 2  Page 7 para 2 |
|  | 11B |  | If blinded, method of blinding and description of the similarity of interventions† | Page 7 para2 |
| Statistical methods† | 12 | Statistical methods used to compare groups for primary outcome(s); methods for additional analyses, such as subgroup analyses and adjusted analyses | When applicable, details of whether and how the clustering by care providers or centers was addressed | Page 7 para 3 & 4 |
| **Results** |  |  |  |  |
| Participant flow† | 13 | Flow of participants through each stage (a diagram is strongly recommended)---specifically, for each group, report the numbers of participants randomly assigned, receiving intended treatment, completing the study protocol, and analyzed for the primary outcome; describe deviations from study as planned, together with reasons | The number of care providers or centers performing the intervention in each group and the number of patients treated by each care provider or in each center | Page 24 figure 1  Page 8 para 4 |
| Implementation of intervention† | New item |  | Details of the experimental treatment and comparator as they were implemented | Page 18 Table 2  Page 8 para 6 |
| Recruitment | 14 | Dates defining the periods of recruitment and follow-up |  | Page 8 para 4 |
| Baseline data† | 15 | Baseline demographic and clinical characteristics of each group | When applicable, a description of care providers (case volume, qualification, expertise, etc.) and centers (volume) in each group | Page 17 Table1  Page 8 para 5 |
| Numbers analyzed | 16 | Number of participants (denominator) in each group included in each analysis and whether analysis was by “intention-to-treat”; state the results in absolute numbers when feasible (e.g., 10/20, not 50%) |  | Page 7 para 3  Page 8 para 4 |
| Outcomes and estimation | 17 | For each primary and secondary outcome, a summary of results for each group and the estimated effect size and its precision (e.g., 95% confidence interval) |  | Page 9  Table 3-5 |
| Ancillary analyses | 18 | Address multiplicity by reporting any other analyses performed, including subgroup analyses and adjusted analyses, indicating those prespecified and those exploratory |  | Page 10 para 4 |
| Adverse events | 19 | All important adverse events or side effects in each intervention group |  | Page 10 para 3 |
| **Discussion** |  |  |  |  |
| Interpretation† | 20 | Interpretation of the results, taking into account study hypotheses, sources of potential bias or imprecision, and the dangers associated with multiplicity of analyses and outcomes | In addition, take into account the choice of the comparator, lack of or partial blinding, and unequal expertise of care providers or centers in each group | Page 10-11 |
| Generalizability† | 21 | Generalizability (external validity) of the trial findings | Generalizability (external validity) of the trial findings according to the intervention, comparators, patients, and care providers and centers involved in the trial | Page 12 para 2 |
| Overall evidence | 22 | General interpretation of the results in the context of current evidence |  | Page 11-12 |

*Additions or modifications to the CONSORT checklist. CONSORT = Consolidated Standards of Reporting Trials.

†This item was modified in the 2007 revised version of the CONSORT checklist.
