# Supplementary material for: Economic Evaluation of a General Hospital Unit for Older People with Delirium and Dementia (TEAM Randomised Controlled Trial)
Source: PLoS One. 2015 Dec 18;10(12):e0140662. doi: 10.1371/journal.pone.0140662 (PMC4687694; doi:10.1371/journal.pone.0140662)
Supplement: S2 Appendix — (DOCX) [file pone.0140662.s003.docx]

**S2 Appendix. Summary of resource-use parameters obtained in the TEAM trial**

|  | | | |
| --- | --- | --- | --- |
| **Service** | **Service parameter/code**^a^ | **Resource-use source** | **Unit cost source**^a^ |
| *Secondary care* |  |  |  |
| Inpatient and day case | Start of episode (date), end of episode (date), episode number, spell number, primary diagnosis (ICD-10 code and description), co-morbidities (ICD-10 code and description), procedures (OPCS-4 code and description), HRG-4 code, source of admission, method of admission, specialty on admission, method of discharge, destination of discharge, site code. | Patient Administration System (PAS) | NHS reference costs 2011/12  (using HRG-4 codes) |
| Outpatient care | Start of episode (date), end of episode (date), type of visit, location, description, Treatment Function Code (TFC), TFC description, attendance status. | Patient Administration System (PAS) | NHS reference costs 2011/12  (Using TFCs) |
| Intensive care | Start of episode (date), end of episode (date), level of care. | Patient Administration System (PAS) | NHS reference costs 2011/12 |
| *Primary care*^b^ |  |  |  |
| Event | Date of event, place of event, type of event, provider, free-text. | GP EAR’s system | PSSRU 2012  NHS wage schedule |
|  |  |  |  |
| Medication and wound dressings | Date of issue, rubric (name of drug), dosage, preparation, acute/repeat | GP EAR’s system | BNF 2012  C&D 2012 |
| *Ambulance service* |  |  |  |
|  | Date and time of event, call stop reason, call sign, resource type, hospital attended, clinic/ward attended by resource, primary complaint, dispatch code, government standard at time of call, time from call until arrival on scene, time at scene. | Caller Aided Dispatch (CAD) system, Patient Record Forms (PRF’s) | NHS reference costs 2011/12 |
| *Mental health care* |  |  |  |
|  | Activity date, activity type, activity code, specialty code, Team/Ward type. | CSE Healthcare RiO | NHS reference costs 2011/12 |
| *Social care* |  |  |  |
|  | Start date, end date, category of contact , service description, reason for referral, outcome of assessment, source | OLM Care First (City)  Corelogic Frameworki (County) | PSSRU 2012 |

^a^If unit costs were sourced from a reference pre-2012 then these costs were standardised to 2012 prices using the Hospital & Community Health Services (HCHS) index [PSSRU 2010 and Curtis 2012] for annual price inflation in the NHS.

^b^Records extracted included consultations, procedures, telephone calls, home visits, administrative tasks, tests ordered and test results received. The protocol for identifying a participant’s GP practice, recruiting practices to the study, identifying participants, extracting data, the anonymisation process and attaching unit costs, and other details of primary care resource-use data collection, are available elsewhere [Franklin et. al. 2014] or upon request
