# Supplementary material for: Economic Evaluation of a General Hospital Unit for Older People with Delirium and Dementia (TEAM Randomised Controlled Trial)
Source: PLoS One. 2015 Dec 18;10(12):e0140662. doi: 10.1371/journal.pone.0140662 (PMC4687694; doi:10.1371/journal.pone.0140662)
Supplement: S3 Appendix — (DOCX) [file pone.0140662.s004.docx]

**S3 Appendix: Description and breakdown of HRG codes used in costing of hospital admission data**

| **HRG code^a^** | **description** | **No. of episodes^c^** | **Inpatient episodes^d^** | **day cases^d^** | **Elective^e^** | **non-elective^e^** | **long admissions^f^** | **short admissions^f^** |
| --- | --- | --- | --- | --- | --- | --- | --- | --- |
| AA02A | Intracranial Procedures for Trauma with Diagnosis of Intracranial Injury with CC^b^ | 1 | 1 | 0 | 0 | 1 | 1 | 0 |
| AA03A | Intracranial Procedures for Trauma with Diagnosis of Head Injury or Skull Fracture, with CC | 3 | 3 | 0 | 0 | 3 | 3 | 0 |
| AA12A | Intermediate Intracranial Procedures Except Trauma with Brain Tumours or Cerebral Cysts, with CC | 2 | 2 | 0 | 0 | 2 | 2 | 0 |
| AA13A | Intermediate Intracranial Procedures Except Trauma with Cerebral Degenerations or Miscellaneous Disorders of Nervous System with CC | 1 | 1 | 0 | 0 | 1 | 1 | 0 |
| AA18A | Minor Intracranial Procedures Except Trauma with Brain Tumours or Cerebral Cysts, with CC | 1 | 1 | 0 | 0 | 1 | 1 | 0 |
| AA22A | Non-Transient Stroke or Cerebrovascular Accident, Nervous System Infections or Encephalopathy, with CC | 89 | 78 | 11 | 25 | 64 | 64 | 25 |
| AA23A | Haemorrhagic Cerebrovascular Disorders with CC | 11 | 10 | 1 | 2 | 9 | 7 | 4 |
| AA24A | Brain Tumours or Cerebral Cysts, with CC | 11 | 10 | 1 | 0 | 11 | 9 | 2 |
| AA25A | Cerebral Degenerations or Miscellaneous Disorders of Nervous System, with CC | 67 | 59 | 8 | 1 | 66 | 33 | 34 |
| AA25B | Cerebral Degenerations or Miscellaneous Disorders of Nervous System, without CC | 1 | 1 | 0 | 0 | 1 | 0 | 1 |
| AA26A | Muscular, Balance, Cranial or Peripheral Nerve Disorders, Epilepsy or Head Injury, with CC | 99 | 88 | 11 | 5 | 94 | 54 | 45 |
| AA27A | Medical Care of Patients with Alzheimer's Disease with CC | 20 | 17 | 3 | 0 | 20 | 13 | 7 |
| AA28A | Motor Neuron Disease with CC | 2 | 0 | 2 | 0 | 2 | 0 | 2 |
| AA28B | Motor Neuron Disease without CC | 3 | 3 | 0 | 0 | 3 | 1 | 2 |
| AA29A | Transient Ischaemic Attack with CC | 7 | 6 | 1 | 1 | 6 | 3 | 4 |
| AA31A | Headache, Migraine or Cerebrospinal Fluid Leak, with CC | 8 | 5 | 3 | 0 | 8 | 3 | 5 |
| AB03Z | Complex Pain Procedures | 2 | 1 | 1 | 1 | 1 | 1 | 1 |
| BZ01Z | Enhanced Cataract Surgery | 1 | 0 | 1 | 1 | 0 | 0 | 1 |
| BZ02Z | Phacoemulsification Cataract Extraction and Lens Implant | 11 | 0 | 11 | 11 | 0 | 0 | 11 |
| BZ05Z | Major Oculoplastics Procedures | 1 | 1 | 0 | 1 | 0 | 1 | 0 |
| BZ12Z | Intermediate Cornea or Sclera Procedures | 1 | 1 | 0 | 0 | 1 | 1 | 0 |
| BZ21Z | Major Vitreous Retinal Procedures | 1 | 0 | 1 | 1 | 0 | 0 | 1 |
| BZ24A | Non-Surgical Ophthalmology with length of stay 2 days or more | 2 | 2 | 0 | 0 | 2 | 2 | 0 |
| BZ24C | Non-Surgical Ophthalmology with length of stay 1 day or less | 4 | 1 | 3 | 1 | 3 | 0 | 4 |
| CZ01Y | Minor Mouth or Throat Procedures, 19 years and over without CC | 1 | 0 | 1 | 1 | 0 | 0 | 1 |
| CZ07O | Exteriorisation of Trachea with Major CC | 2 | 2 | 0 | 0 | 2 | 2 | 0 |
| CZ14V | Major Nose Procedures, 19 years and over with CC | 1 | 1 | 0 | 0 | 1 | 1 | 0 |
| CZ21V | Minor Head, Neck and Ear Disorders, with CC | 60 | 49 | 11 | 0 | 60 | 29 | 31 |
| CZ22W | Intermediate Head, Neck and Ear Disorders, with Major CC | 8 | 7 | 1 | 0 | 8 | 4 | 4 |
| CZ23W | Major Head, Neck and Ear Disorders, with Major CC | 1 | 1 | 0 | 0 | 1 | 1 | 0 |
| DZ06Z | Minor Thoracic Procedures | 1 | 1 | 0 | 0 | 1 | 1 | 0 |
| DZ09A | Pulmonary Embolus with Major CC | 5 | 4 | 1 | 0 | 5 | 4 | 1 |
| DZ09B | Pulmonary Embolus with Intermediate CC | 4 | 4 | 0 | 0 | 4 | 4 | 0 |
| DZ11A | Lobar, Atypical or Viral Pneumonia, with Major CC | 135 | 121 | 14 | 0 | 135 | 96 | 39 |
| DZ11B | Lobar, Atypical or Viral Pneumonia, with Intermediate CC | 19 | 19 | 0 | 2 | 17 | 15 | 4 |
| DZ12A | Bronchiectasis with CC | 2 | 2 | 0 | 0 | 2 | 2 | 0 |
| DZ17A | Respiratory Neoplasms with Major CC | 7 | 6 | 1 | 0 | 7 | 4 | 3 |
| DZ17B | Respiratory Neoplasms with Intermediate CC | 1 | 1 | 0 | 0 | 1 | 1 | 0 |
| DZ19A | Other Respiratory Diagnoses with Major CC | 30 | 20 | 10 | 0 | 30 | 12 | 18 |
| DZ19B | Other Respiratory Diagnoses with Intermediate CC | 5 | 4 | 1 | 1 | 4 | 1 | 4 |
| DZ19C | Other Respiratory Diagnoses without CC | 1 | 1 | 0 | 0 | 1 | 1 | 0 |
| DZ21A | Chronic Obstructive Pulmonary Disease or Bronchitis, with length of stay 1 day or less, discharged home | 21 | 13 | 8 | 0 | 21 | 0 | 21 |
| DZ21E | Chronic Obstructive Pulmonary Disease or Bronchitis, with NIV, without Intubation, with Major CC | 1 | 1 | 0 | 0 | 1 | 0 | 1 |
| DZ21H | Chronic Obstructive Pulmonary Disease or Bronchitis, without NIV, without Intubation, with Major CC | 24 | 23 | 1 | 0 | 24 | 20 | 4 |
| DZ21J | Chronic Obstructive Pulmonary Disease or Bronchitis, without NIV, without Intubation, with Intermediate CC | 13 | 13 | 0 | 0 | 13 | 11 | 2 |
| DZ22A | Unspecified Acute Lower Respiratory Infection with Major CC | 39 | 35 | 4 | 0 | 39 | 27 | 12 |
| DZ22B | Unspecified Acute Lower Respiratory Infection with Intermediate CC | 14 | 12 | 2 | 0 | 14 | 9 | 5 |
| DZ23A | Bronchopneumonia with Major CC | 9 | 8 | 1 | 0 | 9 | 6 | 3 |
| DZ24A | Inhalation Lung Injury or Foreign Body, with Major CC | 13 | 13 | 0 | 0 | 13 | 11 | 2 |
| DZ26A | Pneumothorax or Intrathoracic Injuries, with CC | 2 | 1 | 1 | 0 | 2 | 1 | 1 |
| DZ27D | Respiratory Failure without Intubation, with Major CC | 4 | 4 | 0 | 0 | 4 | 2 | 2 |
| DZ27E | Respiratory Failure without Intubation, with Intermediate CC | 1 | 1 | 0 | 0 | 1 | 1 | 0 |
| EA03Z | Pace 1: Single Chamber or Implantable Diagnostic Device | 3 | 3 | 0 | 1 | 2 | 2 | 1 |
| EA05Z | Pace 2: Dual Chamber | 2 | 2 | 0 | 2 | 0 | 1 | 1 |
| EA10Z | Percutaneous Interventions: Balloon Valve Intermediate Interventions and Arterial Duct Closure | 1 | 1 | 0 | 0 | 1 | 1 | 0 |
| EA17Z | Single Cardiac Valve Procedures | 1 | 1 | 0 | 1 | 0 | 1 | 0 |
| EA31Z | Percutaneous Coronary Intervention, 0 to 2 Stents | 2 | 2 | 0 | 2 | 0 | 1 | 1 |
| EA36A | Catheter, 19 years and over | 2 | 2 | 0 | 1 | 1 | 2 | 0 |
| EB01Z | Non-Interventional Acquired Cardiac Conditions | 69 | 55 | 14 | 0 | 69 | 29 | 40 |
| EB03H | Heart Failure or Shock, with CC | 44 | 37 | 7 | 0 | 44 | 30 | 14 |
| EB03I | Heart Failure or Shock, without CC | 9 | 8 | 1 | 0 | 9 | 4 | 5 |
| EB05Z | Cardiac Arrest | 2 | 2 | 0 | 0 | 2 | 1 | 1 |
| EB06Z | Cardiac Valve Disorders | 3 | 2 | 1 | 0 | 3 | 1 | 2 |
| EB07H | Arrhythmia or Conduction Disorders, with CC | 31 | 28 | 3 | 1 | 30 | 17 | 14 |
| EB07I | Arrhythmia or Conduction Disorders, without CC | 10 | 8 | 2 | 0 | 10 | 6 | 4 |
| EB08H | Syncope or Collapse, with CC | 140 | 115 | 25 | 0 | 140 | 54 | 86 |
| EB08I | Syncope or Collapse, without CC | 21 | 16 | 5 | 0 | 21 | 7 | 14 |
| EB10Z | Actual or Suspected Myocardial Infarction | 43 | 39 | 4 | 1 | 42 | 33 | 10 |
| FZ12D | Major General Abdominal Procedures, 19 years and over with Major CC | 1 | 1 | 0 | 0 | 1 | 1 | 0 |
| FZ13C | Minor Therapeutic or Diagnostic General Abdominal Procedures, 19 years and over | 1 | 0 | 1 | 1 | 0 | 0 | 1 |
| FZ18B | Inguinal, Umbilical or Femoral Hernia Procedures, 19 years and over with Intermediate CC | 1 | 1 | 0 | 0 | 1 | 1 | 0 |
| FZ18C | Inguinal, Umbilical or Femoral Hernia Procedures, 19 years and over without CC | 2 | 1 | 1 | 2 | 0 | 0 | 2 |
| FZ31D | Disorders of the Oesophagus, with length of stay 2 days or more, with Major CC | 2 | 2 | 0 | 0 | 2 | 2 | 0 |
| FZ31F | Disorders of the Oesophagus, with length of stay 1 day or less | 1 | 0 | 1 | 0 | 1 | 0 | 1 |
| FZ36D | Intestinal Infectious Disorders, with length of stay 2 days or more, with Major CC | 8 | 8 | 0 | 1 | 7 | 8 | 0 |
| FZ36E | Intestinal Infectious Disorders, with length of stay 2 days or more, without Major CC | 2 | 2 | 0 | 0 | 2 | 2 | 0 |
| FZ36F | Intestinal Infectious Disorders, with length of stay 1 day or less | 2 | 2 | 0 | 0 | 2 | 0 | 2 |
| FZ37F | Inflammatory Bowel Disease, with length of stay 1 day or less | 8 | 5 | 3 | 1 | 7 | 0 | 8 |
| FZ37G | Inflammatory Bowel Disease, with length of stay 2 days or more, with Interventions, with Major CC | 2 | 2 | 0 | 0 | 2 | 2 | 0 |
| FZ37H | Inflammatory Bowel Disease, with length of stay 2 days or more, without Interventions, with Major CC | 12 | 12 | 0 | 0 | 12 | 12 | 0 |
| FZ37J | Inflammatory Bowel Disease, with length of stay 2 days or more, without Interventions, without Major CC | 3 | 3 | 0 | 0 | 3 | 3 | 0 |
| FZ38D | Gastrointestinal Bleed, with length of stay 2 days or more, with Major CC | 8 | 8 | 0 | 0 | 8 | 8 | 0 |
| FZ38E | Gastrointestinal Bleed, with length of stay 2 days or more, without Major CC | 2 | 2 | 0 | 0 | 2 | 2 | 0 |
| FZ38F | Gastrointestinal Bleed, with length of stay 1 day or less | 15 | 10 | 5 | 0 | 15 | 0 | 15 |
| FZ39D | Hernia Disorders, with length of stay 2 days or more, with Major CC | 1 | 1 | 0 | 0 | 1 | 1 | 0 |
| FZ39E | Hernia Disorders, with length of stay 2 days or more, without Major CC | 1 | 1 | 0 | 0 | 1 | 1 | 0 |
| FZ39F | Hernia Disorders, with length of stay 1 day or less | 5 | 3 | 2 | 0 | 5 | 0 | 5 |
| FZ41D | Anal Disorders, with length of stay 2 days or more, with Major CC | 1 | 1 | 0 | 0 | 1 | 1 | 0 |
| FZ41F | Anal Disorders, with length of stay 1 day or less | 1 | 1 | 0 | 0 | 1 | 0 | 1 |
| FZ43A | Non-Malignant Stomach or Duodenum Disorders, with length of stay 2 days or more, with Major CC | 6 | 6 | 0 | 0 | 6 | 6 | 0 |
| FZ43B | Non-Malignant Stomach or Duodenum Disorders, with length of stay 2 days or more, without Major CC | 3 | 3 | 0 | 0 | 3 | 3 | 0 |
| FZ43C | Non-Malignant Stomach or Duodenum Disorders, with length of stay 1 day or less | 7 | 5 | 2 | 1 | 6 | 0 | 7 |
| FZ45A | Non-Malignant Large Intestinal Disorders, with length of stay 2 days or more, with Major CC | 4 | 4 | 0 | 0 | 4 | 4 | 0 |
| FZ45B | Non-Malignant Large Intestinal Disorders, with length of stay 2 days or more, without Major CC | 4 | 4 | 0 | 0 | 4 | 4 | 0 |
| FZ45C | Non-Malignant Large Intestinal Disorders, with length of stay 1 day or less | 8 | 7 | 1 | 0 | 8 | 0 | 8 |
| FZ46A | Malignant Large Intestinal Disorders, with length of stay 2 days or more, with Major CC | 1 | 1 | 0 | 0 | 1 | 1 | 0 |
| FZ47A | Non-Malignant General Abdominal Disorders, with length of stay 2 days or more, with Major CC | 10 | 10 | 0 | 0 | 10 | 10 | 0 |
| FZ47B | Non-Malignant General Abdominal Disorders, with length of stay 2 days or more, without Major CC | 4 | 4 | 0 | 1 | 3 | 4 | 0 |
| FZ47C | Non-Malignant General Abdominal Disorders, with length of stay 1 day or less | 18 | 10 | 8 | 0 | 18 | 0 | 18 |
| FZ48A | Malignant General Abdominal Disorders, with length of stay 2 days or more, with Major CC | 1 | 1 | 0 | 0 | 1 | 1 | 0 |
| FZ49A | Nutritional Disorders, with length of stay 2 days or more, with Major CC | 3 | 3 | 0 | 0 | 3 | 3 | 0 |
| FZ49C | Nutritional Disorders, with length of stay 1 day or less | 3 | 2 | 1 | 0 | 3 | 0 | 3 |
| FZ51Z | Diagnostic Colonoscopy, 19 years and over | 3 | 0 | 3 | 2 | 1 | 0 | 3 |
| FZ53Z | Therapeutic Colonoscopy, 19 years and over | 1 | 0 | 1 | 1 | 0 | 0 | 1 |
| FZ54Z | Diagnostic Flexible Sigmoidoscopy, 19 years and over | 3 | 0 | 3 | 1 | 2 | 0 | 3 |
| FZ55Z | Diagnostic Flexible Sigmoidoscopy with Biopsy, 19 years and over | 4 | 0 | 4 | 1 | 3 | 0 | 4 |
| FZ59Z | Intermediate Upper GI Tract Procedures, 19 years and over | 1 | 0 | 1 | 0 | 1 | 0 | 1 |
| FZ60Z | Diagnostic Endoscopic Upper GI Tract Procedures, 19 years and over | 12 | 0 | 12 | 2 | 10 | 0 | 12 |
| FZ61Z | Diagnostic Endoscopic Upper GI Tract Procedures with Biopsy, 19 years and over | 11 | 0 | 11 | 1 | 10 | 0 | 11 |
| FZ64A | Combined Upper and Lower GI Tract Diagnostic Endoscopic Procedures with Biopsy, 19 years and over | 1 | 0 | 1 | 0 | 1 | 0 | 1 |
| FZ65Z | Combined Upper and Lower GI Tract Therapeutic Endoscopic Procedures | 1 | 0 | 1 | 1 | 0 | 0 | 1 |
| FZ70Z | Therapeutic Endoscopic Upper GI Tract Procedures, 19 years and over | 2 | 0 | 2 | 0 | 2 | 0 | 2 |
| FZ76B | Distal Colon Procedures, 19 years and over without Major CC | 1 | 1 | 0 | 1 | 0 | 1 | 0 |
| FZ88A | Insertion of Gastrostomy Tube, 19 years and over | 1 | 0 | 1 | 1 | 0 | 0 | 1 |
| GA10F | Open or Laparoscopic Cholecystectomy, 19 years and over with CC | 1 | 1 | 0 | 1 | 0 | 1 | 0 |
| GB06D | Intermediate Therapeutic Endoscopic Retrograde Cholangiopancreatography, with length of stay 2 days or less | 3 | 0 | 3 | 1 | 2 | 0 | 3 |
| GC15C | Non-Malignant Liver Disorders with Major CCs | 2 | 2 | 0 | 0 | 2 | 1 | 1 |
| GC16B | Non-Malignant Pancreatic or Biliary Disorders, with Severe CCs | 6 | 5 | 1 | 0 | 6 | 5 | 1 |
| GC16C | Non-Malignant Pancreatic or Biliary Disorders, with Major CCs | 6 | 6 | 0 | 0 | 6 | 5 | 1 |
| GC16D | Non-Malignant Pancreatic or Biliary Disorders, without Major CCs | 1 | 1 | 0 | 1 | 0 | 0 | 1 |
| HA11A | Major Hip Procedures for Trauma, Category 2, with Major CC | 2 | 2 | 0 | 0 | 2 | 2 | 0 |
| HA12B | Major Hip Procedures for Trauma, Category 1, with CC | 9 | 9 | 0 | 0 | 9 | 8 | 1 |
| HA12C | Major Hip Procedures for Trauma, Category 1, without CC | 3 | 3 | 0 | 0 | 3 | 3 | 0 |
| HA13A | Intermediate Hip Procedures for Trauma, with Major CC | 7 | 7 | 0 | 0 | 7 | 7 | 0 |
| HA13C | Intermediate Hip Procedures for Trauma, without CC | 3 | 3 | 0 | 0 | 3 | 3 | 0 |
| HA22B | Major Knee Procedures for Trauma, Category 1, with CC | 1 | 1 | 0 | 0 | 1 | 1 | 0 |
| HA23B | Intermediate Knee Procedures for Trauma, Category 2, with CC | 1 | 1 | 0 | 0 | 1 | 1 | 0 |
| HA51Z | Major Hand Procedures for Trauma, Category 2 | 2 | 1 | 1 | 0 | 2 | 1 | 1 |
| HA73C | Minor Elbow and Lower Arm Procedures for Trauma, 19 years and over | 1 | 1 | 0 | 0 | 1 | 1 | 0 |
| HA81A | Sprains, Strains or Minor Open Wounds, with Major CC | 16 | 15 | 1 | 0 | 16 | 11 | 5 |
| HA81B | Sprains, Strains or Minor Open Wounds, with Intermediate CC | 4 | 4 | 0 | 0 | 4 | 4 | 0 |
| HA81C | Sprains, Strains or Minor Open Wounds, without CC | 20 | 15 | 5 | 0 | 20 | 7 | 13 |
| HA83A | Head Injury with Major CC | 6 | 5 | 1 | 0 | 6 | 4 | 2 |
| HA83B | Head Injury with Intermediate CC | 2 | 2 | 0 | 0 | 2 | 1 | 1 |
| HA83C | Head Injury without CC | 9 | 8 | 1 | 0 | 9 | 4 | 5 |
| HA91Z | Hip Trauma Diagnosis without Procedure | 27 | 26 | 1 | 0 | 27 | 19 | 8 |
| HA92Z | Knee Trauma Diagnosis without Procedure | 2 | 2 | 0 | 0 | 2 | 1 | 1 |
| HA93Z | Foot Trauma Diagnosis without Procedure | 1 | 1 | 0 | 0 | 1 | 0 | 1 |
| HA94Z | Arm Trauma Diagnosis without Procedure | 23 | 18 | 5 | 0 | 23 | 13 | 10 |
| HA96Z | Multiple Trauma Diagnoses without Procedure | 1 | 1 | 0 | 0 | 1 | 1 | 0 |
| HA97Z | Other Trauma Diagnosis without Procedure | 8 | 7 | 1 | 0 | 8 | 6 | 2 |
| HB12B | Major Hip Procedures for Non-Trauma, Category 1, with Intermediate CC | 1 | 1 | 0 | 1 | 0 | 1 | 0 |
| HB15D | Minor Hip Procedures for Non-Trauma, Category 2, 19 years and over with CC | 1 | 1 | 0 | 0 | 1 | 1 | 0 |
| HB23B | Intermediate Knee Procedures for Non-Trauma, with CC | 1 | 1 | 0 | 0 | 1 | 0 | 1 |
| HB24B | Minor Knee Procedures for Non-Trauma, Category 2, with CC | 2 | 2 | 0 | 0 | 2 | 1 | 1 |
| HB24C | Minor Knee Procedures for Non-Trauma, Category 2, without CC | 1 | 1 | 0 | 0 | 1 | 0 | 1 |
| HB51Z | Major Hand Procedures for Non-Trauma, Category 2 | 1 | 0 | 1 | 1 | 0 | 0 | 1 |
| HB55C | Minor Hand Procedures for Non-Trauma, Category 2, without CC | 1 | 1 | 0 | 0 | 1 | 1 | 0 |
| HB63Z | Minor Shoulder and Upper Arm Procedures for Non-Trauma | 1 | 1 | 0 | 0 | 1 | 1 | 0 |
| HB71B | Major Elbow and Lower Arm Procedures for Non-Trauma, with CC | 1 | 1 | 0 | 0 | 1 | 1 | 0 |
| HB99Z | Other Procedures for Non-Trauma | 1 | 1 | 0 | 0 | 1 | 1 | 0 |
| HC12Z | Intradural Spine Minor 1 | 2 | 1 | 1 | 0 | 2 | 0 | 2 |
| HC20B | Vertebral Column Injury without Procedure, with CC | 9 | 8 | 1 | 0 | 9 | 5 | 4 |
| HC27B | Degenerative Spinal Conditions with CC | 14 | 13 | 1 | 0 | 14 | 6 | 8 |
| HC27C | Degenerative Spinal Conditions without CC | 3 | 2 | 1 | 0 | 3 | 1 | 2 |
| HC32B | Low Back Pain with CC | 5 | 5 | 0 | 0 | 5 | 4 | 1 |
| HC32C | Low Back Pain without CC | 6 | 6 | 0 | 0 | 6 | 3 | 3 |
| HD21A | Soft Tissue Disorders with Major CC | 24 | 15 | 9 | 2 | 22 | 13 | 11 |
| HD21B | Soft Tissue Disorders with Intermediate CC | 5 | 3 | 2 | 0 | 5 | 1 | 4 |
| HD21C | Soft Tissue Disorders without CC | 1 | 0 | 1 | 1 | 0 | 0 | 1 |
| HD23A | Inflammatory Spine, Joint or Connective Tissue Disorders, with Major CC | 10 | 8 | 2 | 0 | 10 | 6 | 4 |
| HD23C | Inflammatory Spine, Joint or Connective Tissue Disorders, without CC | 1 | 1 | 0 | 0 | 1 | 1 | 0 |
| HD24A | Non-Inflammatory Bone or Joint Disorders, with Major CC | 6 | 5 | 1 | 0 | 6 | 4 | 2 |
| HD24B | Non-Inflammatory Bone or Joint Disorders, with Intermediate CC | 4 | 3 | 1 | 0 | 4 | 1 | 3 |
| HD25A | Infections of Bones or Joints, with Major CC | 1 | 1 | 0 | 0 | 1 | 1 | 0 |
| HD26A | Musculoskeletal Signs and Symptoms, with Major CC | 14 | 13 | 1 | 0 | 14 | 5 | 9 |
| HD26B | Musculoskeletal Signs and Symptoms, with Intermediate CC | 13 | 11 | 2 | 0 | 13 | 4 | 9 |
| HD39A | Pathological Fractures with Major CC | 2 | 1 | 1 | 1 | 1 | 1 | 1 |
| HD39B | Pathological Fractures with Intermediate CC | 1 | 0 | 1 | 1 | 0 | 0 | 1 |
| HD40B | Malignancy of Bone or Connective Tissue, with Intermediate CC | 1 | 1 | 0 | 0 | 1 | 1 | 0 |
| HR04B | Reconstruction Procedures Category 3, with CC | 1 | 1 | 0 | 1 | 0 | 1 | 0 |
| HR05Z | Reconstruction Procedures Category 2 | 1 | 1 | 0 | 1 | 0 | 1 | 0 |
| JA24B | Unilateral Intermediate Breast Procedures with Intermediate CC | 1 | 1 | 0 | 1 | 0 | 0 | 1 |
| JB21A | Other Burn without Other Procedure, with Major CC | 2 | 1 | 1 | 0 | 2 | 1 | 1 |
| JC03B | Major Skin Procedures Category 1, with Intermediate CC | 2 | 2 | 0 | 1 | 1 | 1 | 1 |
| JC04A | Intermediate Skin Procedures Category 2, with Major CC | 3 | 3 | 0 | 1 | 2 | 3 | 0 |
| JC04B | Intermediate Skin Procedures Category 2, with Intermediate CC | 1 | 1 | 0 | 1 | 0 | 1 | 0 |
| JC15Z | Skin Therapies Level 3 | 1 | 0 | 1 | 0 | 1 | 0 | 1 |
| JD01A | Major Skin Disorders Category 2, with Major CC | 3 | 3 | 0 | 0 | 3 | 2 | 1 |
| JD03A | Intermediate Skin Disorders Category 2, with Major CC | 21 | 19 | 2 | 0 | 21 | 13 | 8 |
| JD03B | Intermediate Skin Disorders Category 2, with Intermediate CC | 5 | 5 | 0 | 1 | 4 | 3 | 2 |
| JD03C | Intermediate Skin Disorders Category 2, without CC | 2 | 1 | 1 | 0 | 2 | 1 | 1 |
| JD04A | Intermediate Skin Disorders Category 1, with Major CC | 6 | 4 | 2 | 0 | 6 | 2 | 4 |
| JD04C | Intermediate Skin Disorders Category 1, without CC | 1 | 0 | 1 | 0 | 1 | 0 | 1 |
| KA06A | Non-Pituitary Neoplasia and Hypoplasia, with CC | 3 | 3 | 0 | 0 | 3 | 3 | 0 |
| KA08Z | Other Endocrine Disorders | 3 | 3 | 0 | 0 | 3 | 3 | 0 |
| KB01A | Diabetes with Hypoglycaemic Disorders, 70 years and over | 9 | 8 | 1 | 0 | 9 | 4 | 5 |
| KB02A | Diabetes with Hyperglycaemic Disorders, 70 years and over with Major CC | 5 | 5 | 0 | 0 | 5 | 1 | 4 |
| KB02B | Diabetes with Hyperglycaemic Disorders, 70 years and over with Intermediate CC | 1 | 0 | 1 | 0 | 1 | 0 | 1 |
| KB02D | Diabetes with Hyperglycaemic Disorders, 69 years and under with Major CC | 3 | 3 | 0 | 0 | 3 | 2 | 1 |
| KB03A | Diabetes with Lower Limb Complications, with Major CC | 1 | 0 | 1 | 0 | 1 | 0 | 1 |
| KB03B | Diabetes with Lower Limb Complications, without Major CC | 4 | 4 | 0 | 0 | 4 | 3 | 1 |
| KC04Z | Inborn Errors of Metabolism | 4 | 2 | 2 | 0 | 4 | 2 | 2 |
| KC05A | Fluid and Electrolyte Disorders, 70 years and over with Major CC | 24 | 21 | 3 | 2 | 22 | 15 | 9 |
| KC05B | Fluid and Electrolyte Disorders, 70 years and over with Intermediate CC | 7 | 6 | 1 | 0 | 7 | 2 | 5 |
| LA04D | Kidney or Urinary Tract Infections, with length of stay 2 days or more, with Major CC | 186 | 186 | 0 | 1 | 185 | 186 | 0 |
| LA04E | Kidney or Urinary Tract Infections, with length of stay 2 days or more, with Intermediate CC | 28 | 28 | 0 | 0 | 28 | 28 | 0 |
| LA04G | Kidney or Urinary Tract Infections, with length of stay 1 day or less | 138 | 105 | 33 | 1 | 137 | 0 | 138 |
| LA07E | Acute Kidney Injury without Interventions, with Major CC | 20 | 18 | 2 | 0 | 20 | 14 | 6 |
| LA07G | Acute Kidney Injury without Interventions, with Intermediate CC | 12 | 8 | 4 | 0 | 12 | 8 | 4 |
| LA08A | Chronic Kidney Disease with length of stay 2 days or more, with Major CC | 2 | 2 | 0 | 0 | 2 | 2 | 0 |
| LA08F | Chronic Kidney Disease with length of stay 1 day or less, not associated with Renal Dialysis | 1 | 1 | 0 | 0 | 1 | 0 | 1 |
| LA09E | General Renal Disorders with length of stay 2 days or more, with Major CC | 4 | 4 | 0 | 0 | 4 | 4 | 0 |
| LB06D | Kidney, Urinary Tract or Prostate Neoplasms, with length of stay 2 days or more, with Major CC | 5 | 5 | 0 | 0 | 5 | 5 | 0 |
| LB06G | Kidney, Urinary Tract or Prostate Neoplasms, with length of stay 1 day or less | 1 | 1 | 0 | 1 | 0 | 0 | 1 |
| LB13A | Major Endoscopic Bladder Procedures with CC | 2 | 2 | 0 | 2 | 0 | 2 | 0 |
| LB15E | Minor Bladder Procedures, 19 years and over | 17 | 12 | 5 | 2 | 15 | 0 | 17 |
| LB16A | Urinary Incontinence or Other Urinary Problems, with Major CC | 14 | 12 | 2 | 1 | 13 | 6 | 8 |
| LB16B | Urinary Incontinence or Other Urinary Problems, with Intermediate CC | 1 | 1 | 0 | 0 | 1 | 1 | 0 |
| LB16C | Urinary Incontinence or Other Urinary Problems, without CC | 2 | 2 | 0 | 1 | 1 | 1 | 1 |
| LB18Z | Attention to Suprapubic Bladder Catheter | 2 | 1 | 1 | 0 | 2 | 0 | 2 |
| LB20A | Infection or Mechanical Problems Related to Genito-Urinary Prostheses, Implants or Grafts, with CC | 2 | 2 | 0 | 0 | 2 | 2 | 0 |
| LB25B | Transurethral Prostate Resection Procedures with Intermediate CC | 1 | 1 | 0 | 1 | 0 | 1 | 0 |
| LB27Z | Minor Endoscopic Prostate or Bladder Neck Procedures (Male) | 1 | 0 | 1 | 1 | 0 | 0 | 1 |
| LB28A | Non-Malignant Prostate Disorders with CC | 1 | 1 | 0 | 0 | 1 | 1 | 0 |
| LB38A | Unspecified Haematuria with Major CC | 3 | 2 | 1 | 1 | 2 | 2 | 1 |
| LB38B | Unspecified Haematuria without Major CC | 2 | 2 | 0 | 0 | 2 | 2 | 0 |
| LB56A | Minor Penis Procedures, 19 years and over | 1 | 0 | 1 | 0 | 1 | 0 | 1 |
| LB72A | Diagnostic Flexible Cystoscopy, 19 years and over | 7 | 0 | 7 | 7 | 0 | 0 | 7 |
| MB03A | Uterus Disorders (including Fibroids), Menstrual Disorders or Endometriosis, with CC | 2 | 2 | 0 | 0 | 2 | 1 | 1 |
| QZ15B | Therapeutic Endovascular Procedures with Intermediate CC | 7 | 6 | 1 | 2 | 5 | 5 | 2 |
| QZ15C | Therapeutic Endovascular Procedures without CC | 1 | 1 | 0 | 1 | 0 | 1 | 0 |
| QZ17A | Non-Surgical Peripheral Vascular Disease with Major CC | 2 | 2 | 0 | 1 | 1 | 2 | 0 |
| QZ17B | Non-Surgical Peripheral Vascular Disease with Intermediate CC | 2 | 2 | 0 | 0 | 2 | 2 | 0 |
| QZ17C | Non-Surgical Peripheral Vascular Disease without CC | 1 | 1 | 0 | 0 | 1 | 1 | 0 |
| QZ20Z | Deep Vein Thrombosis | 10 | 10 | 0 | 0 | 10 | 7 | 3 |
| RC16Z | Minor Vascular Interventional Radiology Procedures | 2 | 2 | 0 | 0 | 2 | 2 | 0 |
| SA01D | Aplastic Anaemia with CC | 2 | 1 | 1 | 1 | 1 | 1 | 1 |
| SA02D | Coagulation Defect with CC | 1 | 1 | 0 | 0 | 1 | 1 | 0 |
| SA04D | Iron Deficiency Anaemia with CC | 4 | 3 | 1 | 0 | 4 | 2 | 2 |
| SA04F | Iron Deficiency Anaemia without CC | 2 | 1 | 1 | 0 | 2 | 0 | 2 |
| SA05D | Megaloblastic Anaemia with CC | 2 | 2 | 0 | 0 | 2 | 1 | 1 |
| SA06D | Myelodysplastic Syndrome with CC | 9 | 9 | 0 | 0 | 9 | 6 | 3 |
| SA09D | Other Red Blood Cell Disorders with CC | 5 | 4 | 1 | 0 | 5 | 2 | 3 |
| SA09F | Other Red Blood Cell Disorders without CC | 9 | 7 | 2 | 0 | 9 | 5 | 4 |
| SA30Z | Plasma Cell Disorders | 1 | 1 | 0 | 0 | 1 | 0 | 1 |
| SA33Z | Diagnostic Bone Marrow Extraction | 1 | 0 | 1 | 1 | 0 | 0 | 1 |
| SB97Z | Same Day Chemotherapy Admission or Attendance | 1 | 0 | 1 | 1 | 0 | 0 | 1 |
| VA10A | Multiple Trauma Diagnoses score <=23, with no Interventions | 1 | 1 | 0 | 0 | 1 | 1 | 0 |
| VA10B | Multiple Trauma Diagnoses score 24-32, with no Interventions | 1 | 0 | 1 | 0 | 1 | 0 | 1 |
| VA10C | Multiple Trauma Diagnoses score 33-50, with no Interventions | 11 | 7 | 4 | 0 | 11 | 6 | 5 |
| VA10D | Multiple Trauma Diagnoses score >=51, with no Interventions | 10 | 9 | 1 | 2 | 8 | 9 | 1 |
| VA11C | Multiple Trauma Diagnoses score 33-50, with Interventions score 1-8 | 1 | 1 | 0 | 0 | 1 | 1 | 0 |
| VA12C | Multiple Trauma Diagnoses score 33-50, with Interventions score 9-18 | 1 | 1 | 0 | 0 | 1 | 1 | 0 |
| WA03V | Septicaemia with Major CC | 11 | 9 | 2 | 0 | 11 | 7 | 4 |
| WA03X | Septicaemia with Intermediate CC | 1 | 1 | 0 | 0 | 1 | 1 | 0 |
| WA04S | Acute Febrile Illness with length of stay 4 days or less, with Major CC | 4 | 3 | 1 | 0 | 4 | 0 | 4 |
| WA05Q | Pyrexia of Unknown Origin with length of stay 5 days or more, with CC | 1 | 1 | 0 | 0 | 1 | 1 | 0 |
| WA06W | Other Viral Illness with CC | 1 | 1 | 0 | 0 | 1 | 1 | 0 |
| WA09W | Other Non-Viral Infection with CC | 1 | 1 | 0 | 0 | 1 | 1 | 0 |
| WA11V | Poisoning, Toxic, Environmental and Unspecified Effects, with Major CC | 3 | 3 | 0 | 0 | 3 | 0 | 3 |
| WA11X | Poisoning, Toxic, Environmental and Unspecified Effects, with Intermediate CC | 4 | 4 | 0 | 0 | 4 | 3 | 1 |
| WA12V | Complications of Procedures with Major CC | 3 | 3 | 0 | 0 | 3 | 2 | 1 |
| WA12X | Complications of Procedures with Intermediate CC | 2 | 1 | 1 | 0 | 2 | 1 | 1 |
| WA14A | Procedure Not Carried Out for Medical or Patient Reasons | 5 | 1 | 4 | 3 | 2 | 1 | 4 |
| WA14B | Procedure Not Carried Out for Other or Unspecified Reasons | 3 | 0 | 3 | 3 | 0 | 0 | 3 |
| WA17V | Other Admissions Related to Neoplasms with Major CC | 1 | 1 | 0 | 0 | 1 | 1 | 0 |
| WA18V | Admission for Unexplained Symptoms with Major CC | 125 | 96 | 29 | 0 | 125 | 47 | 78 |
| WA18X | Admission for Unexplained Symptoms with Intermediate CC | 74 | 64 | 10 | 1 | 73 | 26 | 48 |
| WA18Y | Admission for Unexplained Symptoms without CC | 1 | 1 | 0 | 0 | 1 | 0 | 1 |
| WA19W | Abnormal Findings without Diagnosis, with CC | 1 | 1 | 0 | 0 | 1 | 0 | 1 |
| WA20W | Examination, Follow-up or Special Screening, with CC | 3 | 2 | 1 | 0 | 3 | 2 | 1 |
| WA22V | Other Specified Admissions or Counselling, with Major CC | 78 | 71 | 7 | 0 | 78 | 61 | 17 |
| WA22X | Other Specified Admissions or Counselling, with Intermediate CC | 28 | 25 | 3 | 1 | 27 | 20 | 8 |
| WA22Y | Other Specified Admissions or Counselling, without CC | 1 | 0 | 1 | 0 | 1 | 0 | 1 |
| WA23V | Falls without Specific Cause, with Major CC | 98 | 66 | 32 | 0 | 98 | 24 | 74 |
| WA23X | Falls without Specific Cause, with Intermediate CC | 38 | 30 | 8 | 0 | 38 | 10 | 28 |
| WA23Y | Falls without Specific Cause, without CC | 2 | 2 | 0 | 0 | 2 | 1 | 1 |
| WD11Z | All patients 70 years and older with a Mental Health Primary Diagnosis, treated by a Non-Specialist Mental Health Service Provider | 189 | 164 | 25 | 1 | 188 | 116 | 73 |
| WD22Z | All patients between 19 and 69 years with a Mental Health Primary Diagnosis, treated by a Non-Specialist Mental Health Service Provider | 8 | 7 | 1 | 0 | 8 | 4 | 4 |

^a^Healthcare resource group (HRG) classification includes groups of hospital episodes with a similar level of healthcare resource use. This study includes HRG version 4 which is used in NHS reference costs 2011/12 and is the main source of hospital unit costs in this study

^b^CC= critical care

^c^No. of episodes of admitted hospital care (inpatient or day cases) with the corresponding HRG code

^d^No. of inpatient (length of stay ≥1 night) and day care (no overnight admission) episodes with the corresponding HRG code

^e^No. of elective (planned) and non-elective (emergency) episodes of admitted hospital care with the corresponding HRG code

^f^No. of short (overnight admission) and long (length of stay >1 night) episodes of admitted care with corresponding HRG code
