# Supplementary material for: Economic Evaluation of a General Hospital Unit for Older People with Delirium and Dementia (TEAM Randomised Controlled Trial)
Source: PLoS One. 2015 Dec 18;10(12):e0140662. doi: 10.1371/journal.pone.0140662 (PMC4687694; doi:10.1371/journal.pone.0140662)
Supplement: S4 Appendix — Proportions missing between groups for variables of interest (Table A). Logistic regression: predictors of missing value for baseline EQ-5D, 599 observations (Table B). Logistic regression: predictors of missing value for follow-up EQ-5D,460 observations (Table C). Logistic regression: predictors of missing value for follow-up proxy EQ-5D, 460 observations (Table D). Logistic regression: predictors of missing value for follow-up Barthel ADL, 460 observations (Table E). Logistic regression: predictors of missing value for follow-up DEMQOL, 460 observations (Table F). (DOCX) [file pone.0140662.s005.docx]

**S4 Appendix. Missing data patterns and predictors**

**Table A. Proportions missing between groups for variables of interest**

| Variable | Number of participants with missing value (% in the group) | | P-value |
| --- | --- | --- | --- |
|  | MMHU (309 patients) | Standard care (290 patients) |  |
| Baseline EQ-5D (proxy completed) | 113 (36.6%) | 87 (30.0%) | 0.09 |
| Follow-up EQ-5D (proxy completed)^a^ | 112 (36.2%) | 85 (29.3%) | 0.27 |
| Primary care cost | 68 (22.0%) | 63 (21.7%) | 0.93 |
| Primary care one year pre-admission cost | 77 (24.9%) | 78 (26.9%) | 0.58 |
| Inpatient one year pre-admission cost | 0 (0.0%) | 2 (0.7%) | 0.14 |
| Follow-up DEMQOL^a^ | 131 (42.4%) | 115 (39.7%) | 0.50 |
| Baseline Barthel ADL index | 3 (1.0%) | 2 (0.7%) | 0.70 |
| Follow-up Barthel ADL index^a^ | 56 (18.1%) | 36 (12.4%) | 0.053 |
| Baseline Neuro-Psychiatric Inventory (NPI) index | 79 (25.6%) | 69 (23.8%) | 0.61 |
| Follow-up Neuro-Psychiatric Inventory (NPI) index^a^ | 164 (53.1%) | 153 (52.8%) | 0.94 |

^a^Missing value cases excluding 139 (MMHU: 68) patients dead at follow up.

**Table B. Logistic regression: predictors of missing value for baseline EQ-5D (599 observations)^a^**

| Variable | Odds ratio (95% CI) | p-value |
| --- | --- | --- |
| Age | 1.01 (0.98, 1.04) | 0.45 |
| Sex (female) | 0.86 (0.60, 1.25) | 0.43 |
| Number of medical conditions | 1.11 (1.03, 1.20) | 0.01 |
| Care home residence at baseline | 3.60 (2.43, 5.34) | <0.001 |

**^a^**Prob > chi2 = 0.000, Pseudo R2 = 0.068. Regression error specification test (RESET) for logistic regression model with a chi-squared statistic (poor specification p-value<0.05): p-value= 0.159.

**Table C. Logistic regression: predictors of missing value for follow-up EQ-5D (460 observations)^a^**

| Variable | Odds ratio (95% CI) | p-value |
| --- | --- | --- |
| Age | 0.97 (0.94, 0.99) | 0.04 |
| Sex (female) | 1.11 (0.76, 1.62) | 0.59 |
| Number of medical conditions | 0.97 (0.89, 1.06) | 0.50 |
| Care home residence at baseline | 1.64 (1.03, 2.59) | 0.04 |

**^a^**Excluding patients dead at follow-up. Prob > chi2 = 0.049, Pseudo R2 = 0.015. RESET test for model specification: p-value= 0.958.

**Table D. Logistic regression: predictors of missing value for follow-up proxy EQ-5D (460 observations)^a^**

| Variable | Odds ratio (95% CI) | p-value |
| --- | --- | --- |
| Age | 0.93 (0.90, 0.96) | <0.001 |
| Sex (female) | 1.23 (0.84, 1.82) | 0.29 |
| Number of medical conditions | 1.06 (0.97, 1.16) | 0.17 |
| Care home residence at baseline | 0.59 (0.36, 0.97) | <0.001 |

**^a^**Excluding patients dead at follow-up. Prob > chi2 = 0.000, Pseudo R2 = 0.044. RESET test for model specification: p-value= 0.392.

**Table E. Logistic regression: predictors of missing value for follow-up Barthel ADL (460 observations)^a^**

| Variable | Odds ratio (95% CI) | p-value |
| --- | --- | --- |
| Age | 0.95 (0.92, 0.98) | 0.003 |
| Sex (female) | 1.24 (0.77, 1.98) | 0.37 |
| Number of medical conditions | 1.02 (0.92, 1.14) | 0.66 |
| Care home residence at baseline | 0.56 (0.30, 1.07) | 0.08 |

**^a^**Excluding patients dead at follow-up. Prob > chi2 = 0.015, Pseudo R2 = 0.027. RESET test for model specification: p-value= 0.829.

**Table F. Logistic regression: predictors of missing value for follow-up DEMQOL (460 observations)^a^**

| Variable | Odds ratio (95% CI) | p-value |
| --- | --- | --- |
| Age | 0.97 (0.94, 1.00) | 0.046 |
| Sex (female) | 1.20 (0.82, 1.74) | 0.35 |
| Number of medical conditions | 0.94 (0.86, 1.02) | 0.17 |
| Care home residence at baseline | 2.02 (1.25, 3.26) | 0.08 |

**^a^**Excluding patients dead at follow-up. Prob > chi2 = 0.004, Pseudo R2 = 0.024. RESET test for model specification: p-value= 0.91.
