# Supplementary material for: Economic Evaluation of a General Hospital Unit for Older People with Delirium and Dementia (TEAM Randomised Controlled Trial)
Source: PLoS One. 2015 Dec 18;10(12):e0140662. doi: 10.1371/journal.pone.0140662 (PMC4687694; doi:10.1371/journal.pone.0140662)
Supplement: S5 Appendix — Baseline characteristics–covariates included in the full-sample adjusted CEA (Table A). Baseline characteristics–covariates included in the complete-case adjusted CEA (Table B). (DOCX) [file pone.0140662.s006.docx]

**S5 Appendix. Baseline characteristics by trial arm**

**Table A. Baseline characteristics – covariates included in the full-sample adjusted CEA**

| Variable | Complete data^a^ | | Imputed data^a^ | |
| --- | --- | --- | --- | --- |
|  | MMHU  (309 patients) | Standard care (290 patients) | MMHU  (309 patients) | Standard care (290 patients) |
| Baseline EQ-5D (217/187^b^) | 0.515 (0.025) | 0.479 (0.026) | 0.478 (0.026) | 0.445 (0.028) |
| Baseline NPI index (230/221) | 31.9 (1.7) | 30.9 (1.7) | 31.9 (1.7) | 30.8 (1.7) |
| Baseline Barthel ADL index (306/288) | 9.0 (0.3) | 8.6 (0.3) | 9.1 (0.3) | 8.6 (0.3) |
| Baseline primary care cost (£) (232/212)^c^ | 326 (20) | 299 (15) | 324 (18) | 301 (15) |
| Baseline inpatient cost (£) (309/288)^c^ | 4614 (419) | 4423 (474) | 4614 (419) | 4438 (475) |
| *Baseline healthcare cost (£)*  *(232/211)* | *6232 (542)* | *5798 (593)* | *6025 (440)* | *5819 (506)* |
| Age | 84.1 (3.5) | 84.2 (4.0) | Data complete | |
| Female | 45.3% | 51.0% |  |  |
| Number of medical conditions | 4.1 (0.1) | 4.3 (0.1) |  |  |
| Baseline care home residence | 28.2% | 20.7% |  |  |
| Baseline outpatient cost (£)^c^ | 579 (938) | 648 (953) |  |  |
| Baseline day-case cost (£)^c^ | 144 (25) | 125 (24) |  |  |
| Baseline critical care cost (£)^c^ | 35 (20) | 37 (19) |  |  |
| Baseline mental health trust cost (£)^c^ | 328 (48) | 270 (38) |  |  |

^a^Mean value (standard error) or percentage of participants with particular characteristic. ^b^Number of participants with complete data for particular variable (MMHU/standard care). ^c^These variables were aggregated to baseline healthcare cost (total one-year pre-admission healthcare cost) which was the covariate in GLM regression models for costs.

**Table B. Baseline characteristics – covariates included in the complete-case adjusted CEA***

|  | MMHU^a^  (109 patients) | Standard care^a^ (100 patients) |
| --- | --- | --- |
| Age | 84.8 (0.55) | 83.5 (0.69) |
| Female | 45.9% | 54.0% |
| Baseline EQ5D | 0.534 (0.037) | 0.508 (0.035) |
| Number of medical conditions | 4.1 (0.2) | 4.0 (0.20) |
| Baseline care home residence | 22.9% | 8.0% |
| Delirium at admission (DRS-R-98 ≥ 18) | 35.8% | 45.6% |
| Severe cognitive impairment (MMSE ≤ 9) | 10.1% | 13.0% |
| Baseline inpatient cost (£)^b^ | 5430 (902) | 4443 (915) |
| Baseline outpatient cost (£)^b^ | 601 (57) | 783 (1020) |
| Baseline day-case cost (£)^b^ | 168 (41) | 166 (52) |
| Baseline critical care cost (£)^b^ | 0 | 50 (31) |
| Baseline mental health trust cost (£)^b^ | 107 (27) | 348 (82) |
| *Baseline secondary and tertiary care cost (£)* | *6306 (923)* | *5790 (961)* |

^a^Mean value (standard error) or percentage of participants with particular characteristic. ^b^These variables were aggregated to baseline secondary and tertiary care cost (total one-year pre-admission cost) which was the covariate in GLM regression models for costs.
