# Supplementary material for: Economic Evaluation of a General Hospital Unit for Older People with Delirium and Dementia (TEAM Randomised Controlled Trial)
Source: PLoS One. 2015 Dec 18;10(12):e0140662. doi: 10.1371/journal.pone.0140662 (PMC4687694; doi:10.1371/journal.pone.0140662)
Supplement: S1 Protocol — Evaluation of a Medical and Mental Health Unit compared with standard care for older people whose emergency admission to an acute general hospital is complicated by concurrent 'confusion': a controlled clinical trial. Acronym: TEAM: Trial of an Elderly Acute care Medical and mental health unit. Trials 2011;12(1):123. (PDF) [file pone.0140662.s007.pdf]

Harwood et al. Trials 2011, 12:123
http://www.trialsjournal.com/content/12/1/123

TRIALS

STUDY PROTOCOL Open Access

Evaluation of a Medical and Mental Health Unit compared with standard care for older people whose emergency admission to an acute general hospital is complicated by concurrent 'confusion': a controlled clinical trial. Acronym: TEAM: Trial of an Elderly Acute care Medical and mental health unit

Rowan H Harwood[1,2*], Sarah E Goldberg[1], Kathy H Whittamore[1,2], Catherine Russell[1,2], John RF Gladman[1], Rob G Jones[3,4], Davina Porock[5], Sarah A Lewis[6], Lucy E Bradshaw[1], Rachel A Elliot[7] and for of Medical Crises in Older People Study Group (MCOP)

Abstract

Background: Patients with delirium and dementia admitted to general hospitals have poor outcomes, and their carers report poor experiences. We developed an acute geriatric medical ward into a specialist Medical and Mental Health Unit over an eighteen month period. Additional specialist mental health staff were employed, other staff were trained in the 'person-centred' dementia care approach, a programme of meaningful activity was devised, the environment adapted to the needs of people with cognitive impairment, and attention given to communication with family carers. We hypothesise that patients managed on this ward will have better outcomes than those receiving standard care, and that such care will be cost-effective.

Methods/design: We will perform a controlled clinical trial comparing in-patient management on a specialist Medical and Mental Health Unit with standard care. Study participants are patients over the age of 65, admitted as an emergency to a single general hospital, and identified on the Acute Medical Admissions Unit as being 'confused'. Sample size is 300 per group. The evaluation design has been adapted to accommodate pressures on bed management and patient flows. If beds are available on the specialist Unit, the clinical service allocates patients at random between the Unit and standard care on general or geriatric medical wards. Once admitted, randomised patients and their carers are invited to take part in a follow up study, and baseline data are collected. Quality of care and patient experience are assessed in a non-participant observer study. Outcomes are ascertained at a follow up home visit 90 days after randomisation, by a researcher blind to allocation. The primary outcome is days spent at home (for those admitted from home), or days spent in the same care home (if admitted from a care home). Secondary outcomes include mortality, institutionalisation, resource use, and scaled outcome measures, including quality of life, cognitive function, disability, behavioural and psychological symptoms, carer strain and carer satisfaction with hospital care. Analyses will comprise comparisons of process, outcomes and costs between the specialist unit and standard care treatment groups.

Trial Registration number: ClinicalTrials.gov: NCT01136148

* Correspondence: rowan.harwood@nuh.nhs.uk
[1]Division of Rehabilitation and Ageing, University of Nottingham, Nottingham NG7 1RD, UK
Full list of author information is available at the end of the article

BioMed Central

© 2011 Harwood et al; licensee BioMed Central Ltd. This is an Open Access article distributed under the terms of the Creative Commons Attribution License (http://creativecommons.org/licenses/by/2.0), which permits unrestricted use, distribution, and reproduction in any medium, provided the original work is properly cited.

Background

Two-thirds of UK National Health Service (NHS) general hospital (i.e. non-psychiatric) beds are occupied by people over 65. Previous reports suggest that 60% of this age group have, or develop, a mental health problem, including dementia (about 30%), delirium (about 20%) and depression (about 30%). About 10% of elderly medical inpatients have significant behavioural disturbance [1].

Ill older people with mental health problems fit uneasily into general hospital services. Presentations can be non-specific (falls, immobility, worsening confusion, not coping). Patients are prone to deterioration and complications. Many general hospital staff feel ill-equipped to assess or manage them. Outcomes are worse (high rates of mortality and care home placement), and length of hospital stay is longer than for people without mental health problems [1]. Relatives are often stressed and dissatified with care [2].

Nationally in the UK, there are some specialist psychiatric liaison services for older people, and a few joint medical-psychiatric units. Such services have been advocated recently in policy documents [1,3,4], but little research has been done on the particular needs and problems of this group, or the best way to configure services to address them. The National Dementia Strategy calls for improvements in care for people with dementia admitted to hosptial, but without providing details on how to do this [5].

There is good evidence that co-ordinated, systematic, multidisciplinary care for people with stroke improves outcomes [6]. The approach called 'comprehensive geriatric assessment', including diagnostic, functional, psychological, social and environmental dimensions, improves outcomes for older people with complex health problems [7].

We hypothesised that a specialist unit for people admitted as emergencies to a general hospital with concurrent dementia or delirium would be similarly effective, and cost-effective. We developed a specialist Medical and Mental Health Unit (MMHU) over a period of eighteen months [8], before undertaking an evaluation by controlled clinical trial.

The trial forms part of a National Institute for Health Research programme, Medical Crises in Older People [9]. The first phase comprised recruiting and following up a cohort (or case series) of 250 older people with mental health problems admitted as an emergency to a general hospital. The recruitment procedures and documentation for this study were almost identical to those intended for the trial, representing a pilot study of the methods.

Methods/design

Main research hypothesis

Patients admitted as an emergency to a general hospital with concurrent 'confusion' and managed on a specialist ward (MMHU), will spend more days at home out of the 90 days following randomisation, than those receiving standard care [10].

Secondary hypotheses

Compared with standard care, management on the MMHU will be associated with:

1. lower mortality
2. better quality of life, less behavioural disturbance, and less disability after 90 days
3. fewer readmissions, reduced total hospital stay over 90 days, and fewer new care home placements
4. greater satisfaction with hospital care amongst family carers
5. less carer strain and psychological dysfunction.

Management on the MMHU is cost-effective compared with standard care.

Management on the MMHU is associated with better quality of care and patient experience in a concurrent observational study.

Overview of study design

Potentially suitable patients are referred to MMHU by clinical staff on the Acute Medical Admissions Units, within 24 hours of admission. MMHU staff record details on a computerised screening log. An algorithm allocates patients either to MMHU or a standard care ward, some at random, others not (Figure 1). If allocated to MMHU the patient is transferred immediately. If allocated standard care, an alternative bed is found by Admissions Unit staff.

Research staff monitor the list of randomly allocated patients, and, as soon as they can be contacted, invite them, and a carer, to take part in the trial. If they agree, baseline data are collected. Satisfaction with care is recorded by the carer one to three weeks after discharge; other outcomes are ascertained by interview 90 days after randomisation. Quality of care and patient experience are assessed by observation. Resource use is collected by questionnaire, and from electronic service records.

The trial flow diagram is given in Figure 2.

Inclusion criteria

The study population comprises patients admitted to the Acute Medical Admissions Unit of a single large teaching hospital (providing sole emergency general medical services for its catchment population), who are aged over 65, and thought to be 'confused' on initial assessment by the clinical team responsible for their care. 'Confusion' is not defined further. This definition is broad and simple enough to allow identification and referral of suitable patients by non-specialist Admissions Unit staff, without causing delay in the admission pathway. In practice, almost all patients identified as 'confused' have delirium and/or dementia.

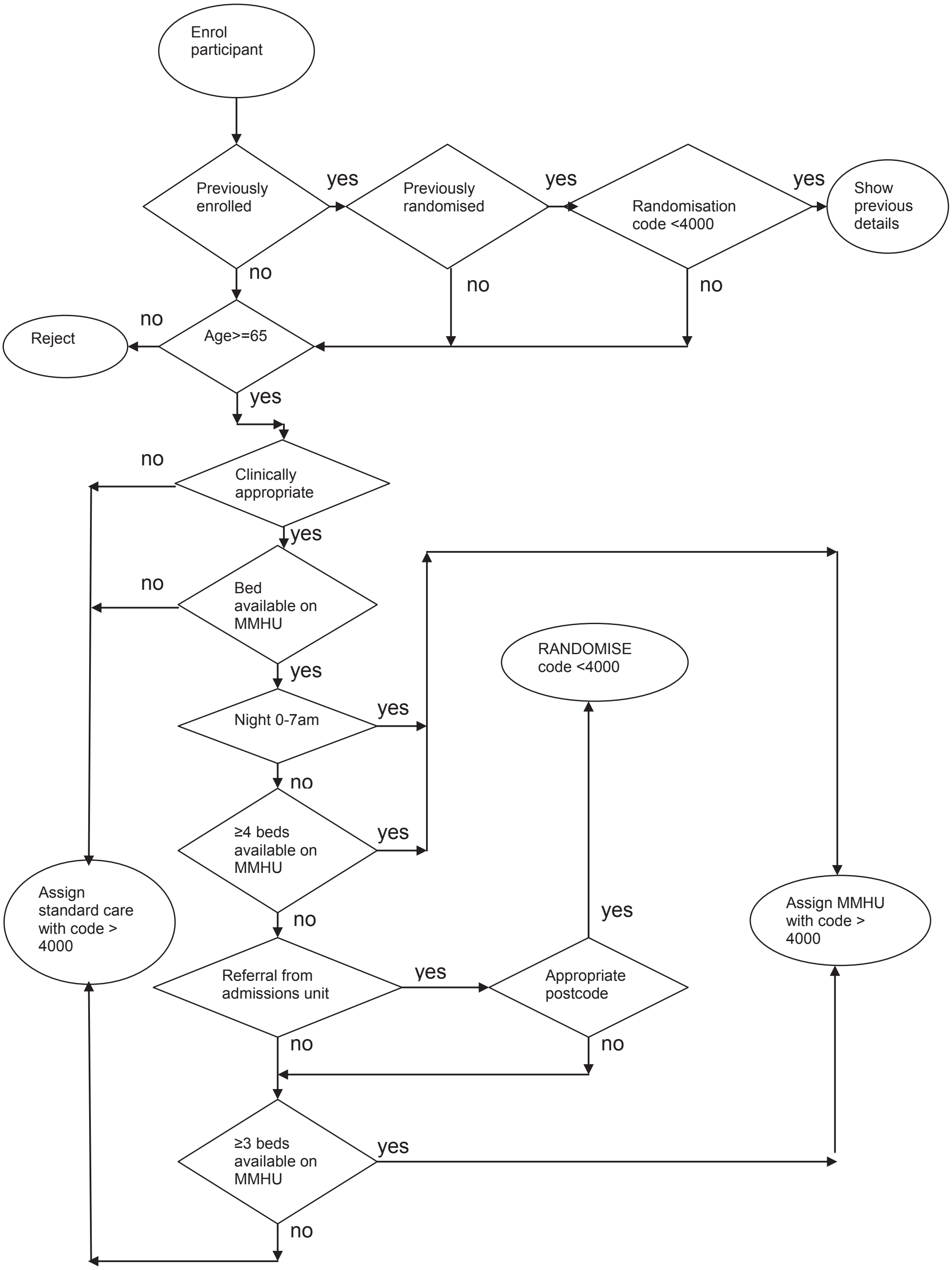

Figure 1 Randomisation and bed management algorithm. Patients with codes <4000 are randomised with stratification according to care home residence or not, and are approached for trial inclusion. Patients with codes >4000 are not randomly allocated, and are not eligible for trial inclusion.

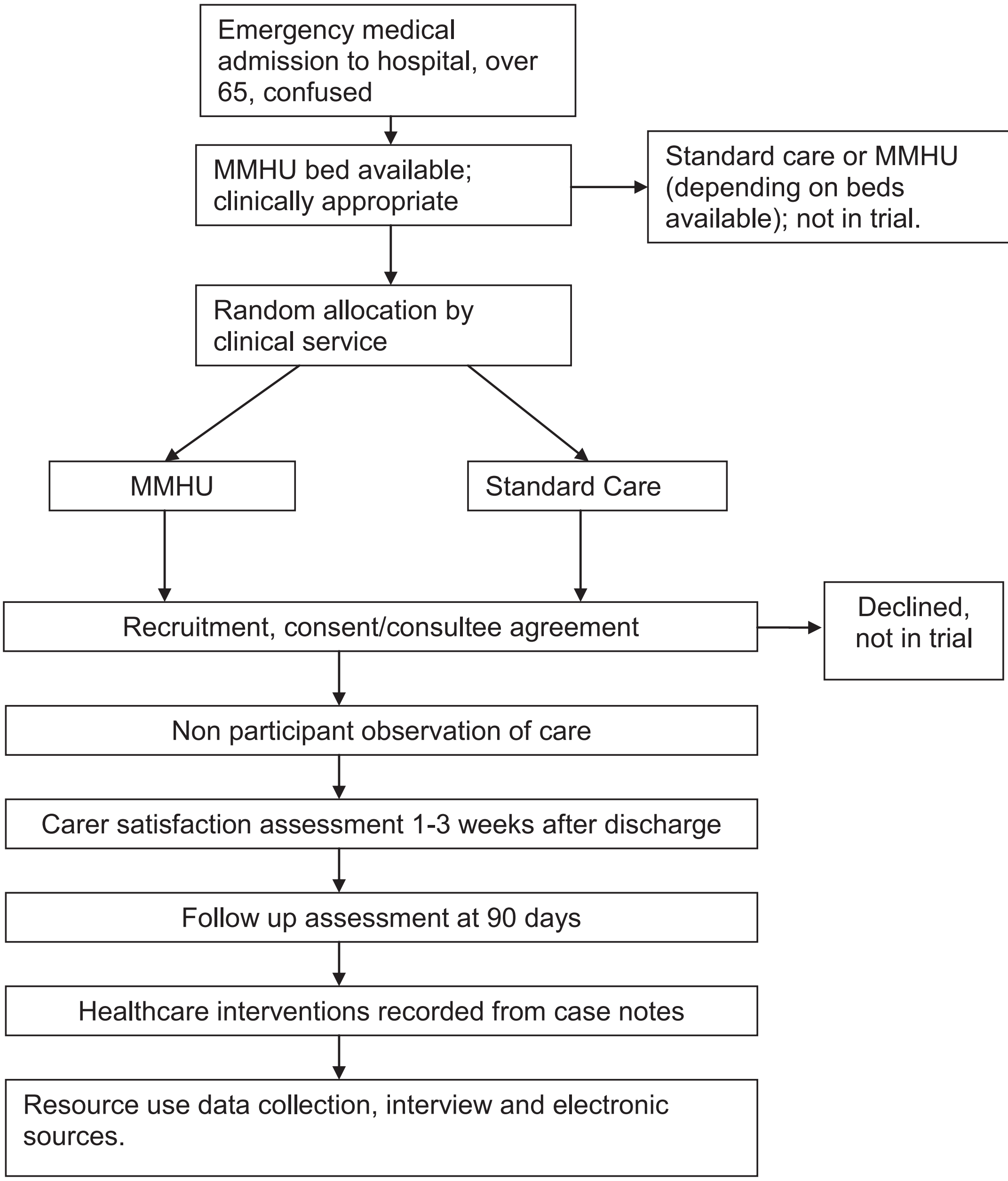

Figure 2 Trial flow diagram.

We also try to recruit a family member or carer participant, where one is available and willing, to act both as an informant, and in order to study impact on carer health. A carer is defined as a non-professional, who sees the patient at least once a week, most weeks, for at least one hour.

Exclusion criteria

- Patients fulfilling clinical exclusion criteria:
  - severely medically ill, requiring intensive monitoring or therapy (critical care), or specialist medical intervention (e.g. severe acute gastrointestinal bleeding, respiratory support)
  - those with an overriding clinical need for another service, such as orthopaedics, or acute stroke
  - acute intoxication or overdose
  - those detained under the Mental Health Act.
- Patients admitted to the MMHU or standard care, who have not been randomised.
- Patients resident outside of Nottingham City or Nottinghamshire County Primary Care Trust (PCT) areas,

for whom data on health and social care resource use could not be ascertained using routine electronic sources. (PCTs are service commissioners in the English NHS).

- Patients and carers unable to speak English, with no available family or other non-professional translator.

Randomisation

Screening log

All referrals are entered on to an internet-based computerised screening log, hosted by the Nottingham University Clinical Trials Unit.

Bed management

We developed an algorithm to manage beds and randomly allocate patients (Figure 1). This was refined over several months of piloting. The precise details reflect local geography, service demands and patient admission rates, taking account of day-to-day variation in both bed availability, and presentation of suitable new patients:

- Randomisation can only take place if there is a bed available on the MMHU (if not, the patient is non-randomly allocated standard care, and is not eligible for trial inclusion)
- The last two beds on the MMHU are always available for randomisation, with the exception of patients referred between midnight and 7 am (a few patients only, to minimise difficult negotiations with bed managers overnight; these are not eligible for trial inclusion)
- If there are four or more beds available on the MMHU, patients are admitted without randomisation (and are not eligible for trial inclusion).
- Patients are also admitted to the MMHU without randomisation (and are not eligible for trial inclusion), if there are three or more empty beds, and patients are:
    - referred from psychiatric wards, following assessment for suitability
    - referred from other hospital wards, following assessment for suitability, but only if not previously randomised to standard care
    - resident outside the Nottingham PCTs areas.

Sequence generation

The randomisation sequence is generated using a computer random number generator, in a 1:1 ratio, and a permuted block design with varying block sizes up to 6, stratified by prior care home residence.

Allocation concealment

The randomisation sequence is concealed from clinical staff who use the computer to allocate patients. However, research staff collecting baseline data are not blind to treatment allocation.

Implementation

A research nurse actively liaises with bed managers during the working day. Out-of-hours a senior clinician (consultant) investigator is always on-call to deal with bed management problems. In practice, the algorithm randomises sufficient patients to recruit our target of between eight and 10 participants per week, whilst remaining acceptable to hospital managers. Readmitted patients are accommodated according to their original allocation.

Treatment definition: Intervention and control groups

The two arms of the trial are:

- Intervention arm: allocation to MMHU
- Control arm: allocation to standard care ward.

Treatment comprises the 'package' of care delivered on the MMHU or standard care wards [8]. These represent complex ('black box') interventions, akin to that provided on stroke units for stroke patients. There is evidence that the overall effect of such units is greater than the sum of identifiable parts [11,12].

The pathway of hospital care for all patients includes admission to an Acute Medical Admissions Unit following referral from a general practitioner, or via the hospital Emergency Department. Following assessment by a senior physician (not necessarily specialising in the care of older people), patients are allocated to a ward.

All wards have access to doctors, ward-based and specialist nurses, physiotherapy, occupational therapy, speech and language therapy, and dietetics. Complex discharge planning and assessment for rehabilitation is supported by a separate multidisciplinary advice team. Mental health support is provided on a consultation basis by psychiatrists from a separate NHS organisation (Mental Health Trust). Social care assessments (provided by local government authorities, not the NHS) are available on request, regardless of ward allocation. All patients, in both settings, are eligible for consideration for standard mental health services, rehabilitation, intermediate and social care.

Standard care

'Standard care' wards include four acute geriatric medical wards, and six general medical wards (with acute medicine, respiratory, diabetes, gastroenterology or rheumatology as their specialist interests). As a matter of policy, the hospital tries to avoid placing confused older medical patients on surgical wards or transferring them (as 'sleepers out') after admission, if there are insufficient medical beds.

Practice on acute geriatric medical wards is based on multidisciplinary comprehensive geriatric assessment, and many staff have considerable experience, and varying degrees of expertise, in the management of delirium and dementia. These wards provide most of the 'standard care' for confused older people.

Medical and Mental Health Unit

The MMHU was developed over 18 months prior to trial commencement. The unit was previously a 28-

bedded acute geriatric medical ward. We have described the development and philosophy of the ward elsewhere [8]. In brief, we enhanced five componants:

- Specialist mental health staff additional to the normal ward complement of medical, nursing and therapy staff, comprising three registered mental health nurses, a specialist mental health occupational therapist (OT), 0.5 whole time equivalent (WTE) specialist physiotherapist, 0.2 WTE speech and langauge therapist, 0.2 WTE additional geriatrician time and 0.1 WTE psychiatrist time, and four unregistered health care assistants, two of whom took on the role of activities co-ordinators. New documentation was introduced for mental health assessments and OT interventions.
- Training for all staff in the philosophy of person-centred dementia care. This emphasises respect for the person with dementia as an individual with a history, values and preferences, and the right to make choices. Confrontation is avoided, and activity and diversion promoted, based on making meaningful connections with the person with dementia's retained abilities.
- A programme of therapeutic and leisure activites was instituted to try to maintain abilities, prevent distress behaviours, and promote night-time rest. Patients are got up and dressed if not too ill.
- The environment was made more appropriate. The ward had to relocate after nine months of development, to one which was longer and with better lay out, when it was realised that sufficient adjustments could not be made in the original one. Noise (such as from radios and equipment alarms) is minimised. Orientation cues, appropriate signage and some safety modifications were made. Bed spaces are personalised.
- A proactive and inclusive approach to family carer-givers is promoted, with active communcation, involvement in decision making, and inclusion in hands on care, if willing.

Contamination

The hospital Trust is developing a strategy of improvement in dementia care, to which members of the MMHU staff have contributed. Other hospital staff are aware of the MMHU, and may have attended teaching or presentations on its work. Some therapy and medical staff work across wards, in particular out-of-hours, or to cover shortages elsewhere, but the additional specialist staff do not. Many hospital-wide policies, such as on nutrition, mental capacity, infection control, end of life care, continence, falls prevention and medicines management are of great relevance to patients with delirium and dementia, and these are promoted on an ongoing basis.

The study therefore represents an evaluation of the additional benefit of care in a geographically-defined unit, with additional staffing and training, and following best practice, beyond that achievable in standard hospital care.

Recruitment and consent

As soon as possible following ward allocation, research staff identify patients who have been randomised. After introduction to the researcher, the patient is assessed for mental capacity to give or withhold consent for participation in the trial. This means understanding, retention, reasoning and communication ability sufficient to decide on willingness to undergo baseline and follow up data collection, and recording use of health and social care. This assessment is done by discussion, using a printed information sheet, supplemented by a simple and short summary, and a checklist of requirements set out in the Mental Capacity Act 2005 [13].

Those having capacity are invited to give written consent to participation. We also ask permission to approach a family member or carer. The family member or carer is given an information sheet and asked to give consent for his or her own involvement in the study.

Most potential participants lack capacity. The procedures set out in section 32 of the Mental Capacity Act are then followed. A family member or carer is asked to act as a 'personal consultee', and asked if they have any reason to believe the patient would not have wanted to take part. If willing, they sign a consultee agreement form. Carers are asked for their own consent to participate.

If there is no contactable carer, or if the carer lacks capacity, the nurse in charge of the ward is asked to act as a 'professional consultee' under section 32(5) of the Mental Capacity Act. If he or she knows no reason why the patient would not want to participate, the patient is included. Most patients in this situation are resident in care homes, and background data are collected from care home staff.

Patients not recruited into the study

Patients not recruited into the study continue with care on the MMHU, or standard care ward, and have no contact with research staff. In practice, ward staff are not aware of who is or is not in the study, even though this is recorded in the case notes, and no distinction is made between recruited and non-recruited patients in care given.

Baseline measurements

Data collection is by interview with a trained researcher. These are either registered nurses or psychology graduates. Information is collected from the patient participant if possible, corroborated by a carer, or taken from family members or carers as informants. Carers are invited to complete a self-report questionnaire, or are interviewed to complete the same information, if they

prefer. Medical and nursing notes are scrutinised for diagnostic, drug and functional information.

Baseline data include:

- Social and demographic information, including co-residence and type of accommodation
- Cognitive impairment (Mini-Mental State Examination [14])
- Delirium diagnosis and severity (Delirium Rating Scale [15])
- Quality of Life (Euroqol EQ5D [16])
- Prior and current physical disability (Barthel index [17])
- Behavioural and psychological symptoms (Neuropsychiatric Inventory [18])
- Medical diagnoses
- Drugs taken on admission
- Illness severity (Modified Early Warning Score [19])
- Hospital admission in the previous year
- Carer relationship, co-residence and amount of care given
- Carer strain index [20]
- Carer psychological distress (GHQ-12 [21]).

Non-participant observation of care

Structured non-participant observations of the experience of care on the MMHU and standard care wards are undertaken using Dementia Care Mapping [22]. For this we include a randomly selected sub-sample of 44 patient participants from the MMHU and 44 patient participants from standard care wards. Patients in the two settings will therefore be matched for disease severity and other factors. Observations are made every five minutes for four to six hours at a time. Field notes are made, and semi-quantified mood and engagement scores, activity codes, and quality of staff interactions with patients are completed.

Recording of health care interventions

Ninety days after randomisation we scrutinise medical, nursing and therapy case notes to ascertain 139 items of assessment, investigation, treatment, information-giving and future care planning information.

Together with the non-participant observer study this will help define if, and how, care delivered in the two settings differs.

Outcome measurements

Definition of primary and secondary outcomes

The primary outcome is number of days spent at home (or in the same care home) in the 90 days following randomisation [10]. This is calculated as 90 days minus days spent dead, in hospital, intermediate care, or a new care home.

It is assumed that discharge home and successfully maintaining it is the goal of health care in this context, taking account of mortality, and readmission, and is a suitable high level summary of the success or failure of hospital acute care.

Secondary outcomes are:

- Mortality
- Quality of life, measured using the dementia-specific Demqol patient and proxy scales [23]; Euroqol EQ5D [16]; and short London Handicap Scale [24]
- Behavioural and psychological symptoms (Neuropsychiatric Inventory [18])
- Carer satisfaction with hospital care (from [2])
- Physical disability (Barthel index [17])
- Cognitive impairment (Mini-Mental State Examination [14])
- Readmission, and total hospital length of stay
- Place of residence
- New care home placement
- Carer strain index [20]
- Carer psychological distress (GHQ-12 [21])
- Health and social care resource use, using the Client Service Receipt Inventory [25] and from routine health and social care records.

Ascertainment of outcomes

Outcomes are measured using:

- a brief telephone call to carer one to three weeks after patient discharge to complete questions on satisfaction with care
- interview of the patient and carer participants at home 90 days (± 7 days) after randomisation
- telephone call to GP or community nurses at 90 days (± 7 days) for community service use
- scrutiny of the hospital computer records for mortality, and readmission
- interrogation of computerised routine health and social care records held by: general and mental health hospitals, general practice, adult social care departments, intermediate and other community based health care, and the ambulance service.

Outcome assessments are done by research staff who were not involved in recruitment or baseline data collection, and who are not deliberately aware of group allocation. They are therefore 'blind' to allocation.

Withdrawal

Patients and carers are free to withdraw from the follow up study without detriment to their care on the MMHU, standard wards, or during follow up. A request to be transferred off the MMHU would be negotiated case by case. Patients allocated standard care have no access to the MMHU.

Data analysis

Power and sample size

We used data from the prior cohort study to estimate sample size and statistical power. The Days at Home

variable has a negatively skewed distribution (median 62 days at home, inter-quartile range 1 to 82 days), with 24% of participants having zero days at home. We assumed that the Mann-Whitney U test would be used to test for differences between groups. We used a bootstrapping simulation method to investigate the power of the study to detect possible effects of the MMHU, on both the probability of zero days at home and numbers of days spent there, under a range of plausible assumptions. A study with 300 participants in each group has 80% power to detect a 5 day difference in days at home, if the proportion of patients with zero days at home is reduced to 20% (a 15% reduction). This represents a reasonable minimum clinically important difference.

Data handling and analysis

Data are double entered into a secure Access database (Microsoft, Redmond, WA).

Descriptive statistics, univariate and multivariate analyses

Baseline and outcome data for each treatment group will be presented as follows: continuous data that are approximately normally distributed will be summarised in terms of the number of observations, mean, standard deviation, minimum and maximum. Skewed data will be transformed to normality, if possible, or otherwise presented in terms of the number of observations, median, lower and upper quartiles, minimum, and maximum. Categorical data will be summarised as frequency counts and percentages.

We will compare baseline differences between groups descriptively to explore whether there are differences in the characteristics of those recruited in the MMHU and standard care groups. The design of this study, with recruitment following randomisation, means that baseline differences may occur that have not arisen by chance, and we will therefore also carry out statistical significance testing of differences at baseline, using parametric or non-parametric tests, as appropriate to the distribution of measured variables.

Analyses of primary and secondary outcomes will be by intention to treat including all those recruited, and will compare differences between MMHU and standard care:

- Mean/median days at home, and total length of hospital stay (and 95% confidence intervals for the differences)
- Proportions dead, at home, in new care homes and readmitted (and 95% confidence intervals for the differences)
- Mean/median scaled outcomes for patients and carers (and 95% confidence intervals for the differences).
- Quantitative and qualitative analysis of the non-participant observer study.
- Proportions of participants receiving various assessment and interventions recorded in the case notes (and difference in proportions with 95% confidence intervals).

Multiple linear, logistic, Poisson and Cox regression will be used, as appropriate to the error distribution of each outcome variable, to model the difference between treatments, adjusting as appropriate for apparent baseline differences, and for other prognostically important variables to improve the precision of effect estimates. We will explore the fit of alternative statistical models for days at home, which will take account of the non-standard distribution of this variable constrained to take values between zero and 90 days, and with an excess of zero values. Non-parametric continuous data will be transformed to normality where possible or otherwise analysed using Mann Whitney U-test. The intervention effect parameter will be presented as an estimate with 95% confidence intervals.

We will use imputation methods for dealing with missing values from those who withdraw, die, or otherwise do not provide follow-up data. We will conduct sensitivity analyses to explore the robustness of the results to alternative assumptions about the randomness of missing data.

Pre-planned subgroup analyses

We will test for interaction between intervention and the following groups. If interaction is present, the results will presented as sub-group analyses, in particular:

- Delirium vs no delirium
- Pre-admission care home vs own home
- Acute geriatric medical ward control group vs other ward
- Died during follow up vs survived
- Index hospital length of stay >5 days vs ≤5 days.

Economic analyses

Costs will be constructed from the perspective of the NHS and personal social services. Ninety days of resource data will be collected for each patient participant. Costs for each participant will be calculated as resource use multiplied by the unit cost of the specific resource, valued using published unit cost data.

A cost-effectiveness analysis comparing the MMHU to standard care will be carried out using standard methods. Incremental cost-effectiveness analysis will be undertaken in the absence of convincing dominance by any treatment alternative. Benefits to the patient participant will be measured with Quality-Adjusted Life Years (QALYs), which will be generated using EQ5D data, assuming homogeneity across treatment groups in any insensitivity of the EQ5D to our patient participants. These will be combined with cost data to generate Incremental Cost-Effectiveness Ratios and Incremental Net Benefit statistics. No discounting will be necessary given the short follow-up time.

Sensitivity analyses and cost-effectiveness acceptability curves will be used to assess uncertainty in the estimated incremental net benefit statistics.

Project management and administration

Project management

Overall supervision of the trial is by the Medical Crises in Older People Programme Management Board, which meets monthly, chaired by the programme Principal Investigator (JG).

In addition, a Trial Steering Committee has been constituted, with an independent chair, three independent professional members (one psychiatrist, one physician, one social scientist), two independent lay members, and three trial investigators, including the statistician.

Day to day management of the project is by a trial manager (SG), supervised by the chief investigator (RH), and assisted by research assistants (KW and CR). The trial is also supported by research assistants employed by the National Institute for Health Research Comprehensive Local Research Network, Mental Health Research Network, and the Trent Dementia Research Network.

The trial sponsor is the University of Nottingham.

Trial documents

The documents to be completed, and by whom, are recorded in table 1.

Ethical approval

The protocol was given a favourable opinion by the Nottingham 1 Research Ethics Committee (reference 10/H0403/16).

Table 1 Trial documentation

Form title	Completed by
Screening log (computerised)	MMHU ward nurses
Capacity assessment form	Researcher
Patient participant information and consent forms	Patient with capacity to consent
Consultee information and agreement forms	Family member of carer (on behalf of patient without capacity to consent)
Carer participant information and consent forms	Family member or carer
Professional information and agreement forms	Nurse in charge of ward (on behalf of patient without capacity to consent and with no contactable carer)
Identifiable data form	Researcher
Patient participant initial data form	Researcher
Carer initial data form	Family member or carer/researcher
Medical data form (from casenotes)	Researcher
Dementia care mapping raw data sheets	Researcher
Patient participant follow-up data form	Researcher
Carer follow-up data form	Family member or carer/researcher
Resource use from computerised records	Health economist
Health care intervention form	Medically qualified researcher

Project milestones

Project milestones are described in table 2.

Discussion

Justification for design

The study design had to accommodate the constraints of an acute medical service very pressed for bed availability, and under rigorous performance management of patient flows, in particular, the government-prescribed maximum four hour Emergency Department wait target. This stipulates that all patients must be assessed, treated, and discharged or transferred from Emergency Departments (ED) within four hours of arrival. This, in turn, puts pressure on Acute Medical Admissions Units, who must have empty beds to accept transfers from ED, and on wards to have capacity to accept patients from Admission Units.

It was, therefore, unacceptable to the clinical service for potential trial participants to remain on the Acute Medical Admissions Unit whilst awaiting research assessment or recruitment procedures, or for there to be more than three empty beds on the MMHU. It had to be possible to admit to the MMHU 24 hours a day and seven days a week, regardless of researcher availability, and to keep the ward full with appropriate patients. Proper time for consultation, consent or consultee agreement for research participation to be given, is necessary for legal and ethical reasons. The consultation and consent process takes longer than the clinical processes for swift bed management. So it was impossible to run a conventional randomised controlled trial with

Table 2 Project milestones

From	To	Activity
2006	2008	Programme planning and funding application
Feb 2009	July 2010	Planning, development and maturation of MMHU intervention
May 2009	June 2010	Recruitment and follow up of cohort (case series), pilot study for the trial
February 2010		Submission to Research Ethics Committee and Hospital Research governance
May 2010	July 2010	Pilot random allocation to MMHU or standard care
July 2010	December 2011	Trial recruitment (possible extension to July 2012)
October 2010	March 2012	Follow up
January 2011	December 2011	Non participant observer study
March 2012	October 2012	Final data cleaning, analysis, write up
April 2013		Service support funding ceases
July 2013		Research funding ceases

recruitment prior to allocation: clinically patients had to be allocated before they could be recruited.

Previous work demonstrated that 30% of acute medical patients over 65 had cognitive impairment, far more than could be accommodated on a single ward. Some allocation mechanism was therefore required by the clinical service. In usual clinical practice, ward allocation is largely driven by bed availability, but in this case the service agreed to allocate suitable patients at random, either to the MMHU, or standard care on another general or geriatric medical ward.

The allocation algorithm ensures that the MMHU remains acceptably full, without compromising recruitment to the trial. Only patients who have been randomly allocated, and their carers, are invited to participate in the trial.

Implications of design

Strictly, the design violates best practice for a randomised controlled trial, since only randomised patients who consent, or have consultee agreement, can be followed up. For this reason, we have chosen to describe it as a 'controlled clinical trial'. However, it approximates to a pragmatic, parallel group, randomised controlled trial.

The main scientific concern about the design is failure to recruit a patient after randomisation. This introduces the potential for bias (for example, if it proves easier to recruit from one setting than the other). Baseline data cannot legally or ethically be collected for research purposes from randomised patients who do not consent (or have consultee agreement) to take part.

Despite the risk of differential recruitment bias, an important consideration is that this design enables us to undertake a trial at all. A conventional randomised controlled design would either have failed because of conflict with the demands of the clinical service, or would have recruited an unrepresentative population. If we attempted to recruit with inadequate consent or consultation, the trial would be illegal, and would be open to bias through high withdrawal rates.

Additional measures to avoid bias

Studying people in urgent care settings unavoidably carries risk of bias. We introduced explicit steps to try to minimise that risk.

Researchers are aware of potential problems with bias, and are trained to adopt a rigorous approach to recruitment, whilst respecting an individual's right not to be involved in research if they so choose, or if circumstances (such as end of life care) make it inappropriate.

Research staff operate shifts to be available when family carers are visiting, to maximise recruitment. A contact log is maintained. The proportion of randomised patients recruited in each setting is monitored closely.

Extensive data is collected at baseline from recruited participants that will enable comparison of groups, and statistical adjustment for baseline imbalances if found.

List of abbreviations

ED: Emergency Department; EQ5D: Euroqol 5-dimension quality of life scale; GHQ-12: General Health Questionnaire 12-item version; GP: General Practitioner (family doctor); MMHU: Medical and Mental Health Unit; NHS: (United Kingdom) National Health Service; OT: Occupational Therapy; PCT: Primary Care Trust (health service commissioner); QALY: Quality Adjusted Life Year; WTE: Whole time equivalent; UK: United Kingdom.

Acknowledgements and funding

The Medical Crises in Older People study group also includes:
Justine Schneider, Simon Conroy, Anthony Avery, Fiona Jurgens, Judy Edmans, Adam Gordon, Bella Robbins, Jane Dyas, Pip Logan, Casey Quinn, Helen Boardman, Matt Franklin.
Dan Simpkins of the Nottingham Clinical Trial Unit devised the allocation algorithm and internet-based randomisation programme. Clinical Service managers Nigel Sturrock, Caron Swinscoe, Steph Beasley-Thomas and John Morrant facilitated implementation of the service and trial design. Clinical staff on the Admission Unit and MMHU, led by ward managers Nikki King, Laura Ray and Naomi Morrison implemented the clinical aspects of the trial. Funding was from the National Institute for Health Research (UK government).

Author details

[1]Division of Rehabilitation and Ageing, University of Nottingham, Nottingham NG7 1RD, UK. [2]Health Care of Older People, Nottingham University Hospitals NHS Trust, Queens Medical Centre, Nottingham NG7 2UH, UK. [3]Division of Psychiatry, University of Nottingham, Nottingham NG7 1RD, UK. [4]Mental Health Services for Older People, Nottinghamshire Healthcare NHS Trust, Queens Medical Centre, Nottingham NG7 2UH,UK. [5]School Of Nursing, State University of New York, Buffalo, Wende Hall 3435, Main Street, Buffalo, NY 14214, USA. [6]Division of Epidemiology and Public Health, University of Nottingham, Nottingham NG7 1RD, UK. [7]School of Pharmacy, University of Nottingham, Nottingham NG7 1RD, UK.

Authors' contributions

RH, JG and RJ conceived the study, obtained grant funding and designed the evaluation. RH, DP and RJ, along with NHS colleagues, conceived, obtained additional funding for, and developed the MMHU. SG, KW and CR operationalised and refined the study design, and obtained ethical and regulatory approvals. SG and KW recruited and followed up most of the patients in the cohort (case series), piloting and refining the recruitment procedures and data collection forms. SL provided statistical advice. RE designed the economic analysis. LB did the power calculations. All authors have read and approved the final manuscript

Competing interests

The authors declare that they have no competing interests.

Received: 25 January 2011 Accepted: 13 May 2011
Published: 13 May 2011

References

1. Royal College of Psychiatrists: *Who cares wins London* 2005.
2. Alzheimer's Society: *Counting the Cost London* 2009.
3. Royal College of Psychiatrists: *Raising the standard London* 2006.
4. Department of Health: *Everybody's Business* London, Department of Health; 2006.
5. Department of Health: *National dementia strategy* London, Department of Health; 2009.
6. Stroke Unit Trialists' Collaboration: **Organised inpatient (stroke unit) care for stroke.** *Cochrane Database of Systematic Reviews* 2001, , **3**: CD000197 [http://www.mrw.interscience.wiley.com/cochrane/clsysrev/articles/CD000197/frame.html].
7. Stuck AE, Siu AL, Wieland GD, Adams J, Rubenstein LZ: **Comprehensive geriatric assessment: a meta-analysis of controlled trials.** *Lancet* 1993, **342**:1032-6.

8. Harwood RH, Porock D, King N, Edwards G, Hammond S, Howe L, Russell C, Howard S, Jones RG, Morrant JD: **Development of a specialist medical and mental health unit for older people in an acute general hospital.** *University of Nottingham Medical Crises in Older People discussion paper series* 2010, **5** [http://www.nottingham.ac.uk/mcop/index.aspx], ISSN 2044-4230 (accessed 28.12.10).
9. [http://nottingham.ac.uk/chs/research/projects/mcop.aspx], (accessed 28.12.10).
10. Gladman JRF, Harwood RH, Conroy SP: **Days at home: an outcome measure in studies of specialist services providing care for older people.** *University of Nottingham Medical Crises in Older People discussion paper series* 2010, **4** [http://www.nottingham.ac.uk/mcop/index.aspx], ISSN 2044-4230 (accessed 28.12.10).
11. Evans A, Perez I, Harraf F, Melbourn A, Steadman J, Donaldson N, Kalra L: **Can differences in management processes explain different outcomes between stroke unit and stroke-team care?** *Lancet* 2001, **358**:1586-92.
12. Langhorne P, Dennis M: **Stroke Units: an evidence based approach.** *BMJ books, London* 1998.
13. Office of Public Sector Information: *Mental Capacity Act 2005* [http://www.opsi.gov.uk/acts/acts2005/ukpga_20050009_en_1].
14. Folstein MF, Folstein SE, McHugh PR: **'Mini-mental state'. A practical method for grading the cognitive state of patients for the clinician.** *Journal of Psychiatric Research* 1975, **12**:189-98.
15. Trzepacz PT, Mittal D, Torres R, Kanary K, Norton J, Jimerson N: **Validation of the Delirium Rating Scale-Revised-98 Comparison With the Delirium Rating Scale and the Cognitive Test for Delirium.** *J Neuropsychiatry Clin Neurosci* 2001, **13**:229-242.
16. EuroQol Group: **EuroQol - A new facility for the measurement of health-related quality of life.** *Health Policy* 1990, **16**:199-208.
17. Wade DT, Collin CL: **The Barthel ADL index: a standard measure of physical disability?** *Int Dis Studies* 1988, **10**:64-67.
18. Cummings JL, Mega M, Gray K, Rosenberg-Thompson S, Carusi DA, Gornbein J: **The Neuropsychiatric Inventory: comprehensive assessment of psychopathology in dementia.** *Neurology* 1994, **44**:2308-14.
19. Subbe CP, Kruger M, Gemmel L: **Validation of a modified Early Warning Score in medical admissions.** *Quarterly Journal of Medicine* 2001, **94**:521-6.
20. Robinson B: **Validation of a Caregiver Strain Index.** *Journal of Gerontology* 1983, **38**:344-348.
21. Goldberg D: **General Health Questionnaire (GHQ-12).** *Windsor: NFER-NELSON* 1992.
22. Brooker D: **Person Centred Dementia Care: Making Services Better (Bradford Dementia Group Good Practice Guides).** *London, Jessica Kingsley* 2006.
23. Smith SC, Lamping DL, Banerjee S, Harwood R, Foley B, Smith P, Cook JC, Murray J, Prince M, Levin E, Mann A, Knapp M: **Measurement of health-related quality of life for people with dementia: development of a new instrument (DEMQOL) and an evaluation of current methodology.** *Health Technology Assessment (Winchester, England)* 2005, **9(10)**:1-93, iii-iv.
24. Harwood RH, Ebrahim S: *The Manual of the London Handicap Scale* Nottingham, University of Nottingham; 1995.
25. Knapp M: *Care Service Receipt Inventory* [http://www.iop.kcl.ac.uk/departments/?locator=355&context=850].

doi:10.1186/1745-6215-12-123
Cite this article as: Harwood *et al.*: Evaluation of a Medical and Mental Health Unit compared with standard care for older people whose emergency admission to an acute general hospital is complicated by concurrent 'confusion': a controlled clinical trial. Acronym: TEAM: Trial of an Elderly Acute care Medical and mental health unit. *Trials* 2011 **12**:123.

Submit your next manuscript to BioMed Central and take full advantage of:

- **Convenient online submission**
- **Thorough peer review**
- **No space constraints or color figure charges**
- **Immediate publication on acceptance**
- **Inclusion in PubMed, CAS, Scopus and Google Scholar**
- **Research which is freely available for redistribution**

Submit your manuscript at
www.biomedcentral.com/submit

BioMed Central
